# Epidermal PPARγ Is a Key Homeostatic Regulator of Cutaneous Inflammation and Barrier Function in Mouse Skin

**DOI:** 10.3390/ijms22168634

**Published:** 2021-08-11

**Authors:** Raymond L. Konger, Ethel Derr-Yellin, Teresa A. Zimmers, Terrence Katona, Xiaoling Xuei, Yunlong Liu, Hong-Ming Zhou, Ed Ronald Simpson, Matthew J. Turner

**Affiliations:** 1 Department of Pathology & Laboratory Medicine, Richard L. Roudebush Veterans Affairs Medical Center, Indianapolis, IN 46202, USA; ederryel@iu.edu (E.D.-Y.); Terrence.Katona@va.gov (T.K.); 2 Department of Pathology & Laboratory Medicine, Indiana University School of Medicine, Indianapolis, IN 46202, USA; 3 The Melvin and Bren Simon Comprehensive Cancer Center, Indiana University School of Medicine, Indianapolis, IN 46202, USA; zimmerst@iu.edu (T.A.Z.); yunliu@iu.edu (Y.L.); 4 Department of Surgery, Indiana University School of Medicine, Indianapolis, IN 46202, USA; xxuei@iu.edu; 5 Department of Biochemistry and Molecular Biology, Indiana University School of Medicine, Indianapolis, IN 46202, USA; 6 Center for Medical Genomics, Indiana University School of Medicine, Indianapolis, IN 46202, USA; edrsimps@iupui.edu; 7 Center for Computational Biology and Bioinformatics, Indiana University School of Medicine, Indianapolis, IN 46202, USA; 8 Department of Medical and Molecular Genetics, Indiana University, Indianapolis, IN 46202, USA; 9 Department of Dermatology, Indiana University School of Medicine, Indianapolis, IN 46202, USA; zhouhm00@yahoo.com (H.-M.Z.); turner41@iu.edu (M.J.T.); 10 Department of BioHealth Informatics, Indiana University-Purdue University Indianapolis, Indianapolis, IN 46202, USA; 11 Department of Dermatology, Richard L. Roudebush Veterans Affairs Medical Center, Indianapolis, IN 46202, USA

**Keywords:** peroxisome proliferator-activated receptor gamma, transcriptomic changes, cutaneous phenotype, asebia

## Abstract

Both agonist studies and loss-of-function models indicate that PPARγ plays an important role in cutaneous biology. Since PPARγ has a high level of basal activity, we hypothesized that epidermal PPARγ would regulate normal homeostatic processes within the epidermis. In this current study, we performed mRNA sequencing and differential expression analysis of epidermal scrapings from knockout mice and wildtype littermates. *Pparg*-/-^epi^ mice exhibited a 1.5-fold or greater change in the expression of 11.8% of 14,482 identified transcripts. Up-regulated transcripts included those for a large number of cytokines/chemokines and their receptors, as well as genes associated with inflammasome activation and keratinization. Several of the most dramatically up-regulated pro-inflammatory genes in *Pparg*-/-^epi^ mouse skin included *Igfl3*, *2610528A11Rik*, and *Il1f6*. RT-PCR was performed from RNA obtained from non-lesional full-thickness skin and verified a marked increase in these transcripts, as well as transcripts for *Igflr1*, which encodes the receptor for Igfl3, and the 2610528A11Rik receptor (*Gpr15*). Transcripts for *Il4* were detected in *Pparg*-/-^epi^ mouse skin, but transcripts for *Il17* and *Il22* were not detected. Down-regulated transcripts included sebaceous gland markers and a number of genes associated with lipid barrier formation. The change in these transcripts correlates with an asebia phenotype, increased transepidermal water loss, alopecia, dandruff, and the appearance of spontaneous inflammatory skin lesions. Histologically, non-lesional skin showed hyperkeratosis, while inflammatory lesions were characterized by dermal inflammation and epidermal acanthosis, spongiosis, and parakeratosis. In conclusion, loss of epidermal *Pparg* alters a substantial set of genes that are associated with cutaneous inflammation, keratinization, and sebaceous gland function. The data indicate that epidermal PPARγ plays an important role in homeostatic epidermal function, particularly epidermal differentiation, barrier function, sebaceous gland development and function, and inflammatory signaling.

## 1. Introduction

Three different isoforms of peroxisome proliferator-activated receptors (PPAR) have been identified: PPARα, PPAR β/δ, and PPARγ. All PPARs belong to the family of type II ligand-activated nuclear transcription factors and bind to DNA at peroxisomal proliferators response elements (PPRE) as a heterodimeric complex with retinoid X receptors [1]. PPARγ was initially associated with its ability to transactivate genes associated with adipogenesis, lipid metabolism, glucose homeostasis and cellular energy balance [1,2]. Whole body deletion of *Pparg* in mice causes lipodystrophy, organomegaly, severe type 2 diabetes and the inability to shift from glucose to lipids as an energy source [3]. Inactivating germline mutations of *PPARG* in humans causes severe lipodystrophy, severe hypertension, diabetes, dyslipidemia, and hepatic steatosis [4]. While synthetic thiazolidinedione-class PPARγ agonists were initially used as anti-diabetic agents, PPARγ ligands have more recently been examined as potential therapeutic or chemoprevention targets for dermatologic conditions, including cancer and inflammatory dermatoses [1,5].

PPARγ is activated by a wide variety of synthetic, dietary, and endogenous ligands [1]. Endogenous ligands include the following lipid species: cyclooxygenase products such as prostaglandin J2 metabolites, lipoxygenase products such as 9- or 13-hydroxyoctadienoic acids and 15-hydroxytetraenoic acid, oxidized glycerophosphocholines, oxidized mono and polyunsaturated fatty acids, and nitroalkene derivatives of linoleic acid [1,6,7,8]. An increasing number of dietary phytochemicals also have PPARγ ligand activity [1,9,10]. Moreover, PPARγ has a high degree of ligand-independent constitutive activity [2]. Thus, the relative abundance of endogenous and dietary PPARγ ligands and the high basal activity suggests that PPARγ may exhibit a high degree of constitutive activity and therefore might be necessary for regulating normal homeostatic cellular functions. This idea is further suggested by the phenotypic changes associated with germline deletion in mice [3].

In skin, PPARγ is widely expressed in keratinocytes of the interfollicular epidermis, as well as the pilosebaceous unit, including hair matrix keratinocytes, dermal papilla cells, inner root sheath cells, and sebocytes [11]. Exogenous PPARγ ligand application is known to suppress keratinocyte growth, induce differentiation, promote lipid synthesis, and enhance epidermal barrier function [6,8]. In cultured sebaceous cells, PPARγ ligands initiate sebocyte differentiation to form sebum lipid droplets [12]. Tissue-specific deletion of *Pparg* in bulb stem cells (Krt15-promoter dependent Cre) resulted in a scarring alopecia and sebaceous gland hypoplasia that resembles human lichen planopilaris [13]. An asebia skin phenotype was also observed in a whole body *Pparg* knockout mouse [11]. In this study, the absence of sebum production was theorized to be responsible for a progressive loss of hair follicles, or alopecia [11]. This idea is supported by other asebia mouse models, which also exhibit alopecia [14,15]. Notably, sebaceous gland hypoplasia in human psoriasis has also been linked to psoriatic alopecia [16].

PPARγ has potent anti-inflammatory activity [1]. There is strong evidence that PPARγ may suppress the inflammation associated with inflammatory dermatoses. Relative to normal human control skin, PPARγ transcripts in psoriatic and atopic lesions are reduced 8- and 3.3-fold, respectively [17]. Another study demonstrated that *PPARG* mRNA is significantly decreased in human lichen planopilaris, a form of scarring (cicatricial) alopecia [13]. In human studies, systemic PPARγ agonists have shown clinical activity in psoriasis [5,18,19], atopic dermatitis [20], and cicatricial alopecia [21]. PPARγ agonists were also effective in a mouse model of psoriasis [22]. However, in mouse models of atopic dermatitis, results have been inconclusive. Several groups reported improvements in eczematous lesions following topical application of PPARγ ligands either alone [23], or in combination with glucocorticoids [24]. However, other groups failed to show therapeutic efficacy of topical PPARγ agonists in mouse models of atopic dermatitis [25,26].

In our studies, we have utilized a keratin 14 (Krt14)-promoter-driven Cre recombinase to delete floxed *Pparg* in mice to examine how the loss of epidermal *Pparg* alters tumorigenesis, contact hypersensitivity, and anti-tumor immune responses [1,27,28,29]. Keratin 14 is expressed in basal keratinocytes throughout the interfollicular and follicular epidermis [30], so this strategy provides an assessment of *Pparg* loss throughout the epidermis. Given that PPARγ has a high level of basal or constitutive activity, we hypothesized that PPARγ might play an important role in regulating homeostatic keratinocyte processes involving lipid metabolism, cellular growth and differentiation, and inflammatory signaling. We therefore performed whole transcriptome sequencing and differential expression analysis and compared the observed changes to our observations of baseline phenotypic changes in mice lacking epidermal *Pparg*. Our data show that loss of epidermal *Pparg* results in a surprising number of changes in the basal transcriptome. In this report, we focus on those transcriptome changes that are associated with sebaceous gland development, lipid barrier formation, keratinocyte differentiation, and inflammation.

## 2. Results

### 2.1. Epidermal Pparg Plays an Important Role in Suppressing Cutaneous Inflammation

B6.129-*Pparg^tm2Rev^*/J mice have two loxP sites flanking exons 1 and 2 of the *Pparg* gene [31]. Cre-mediated excision of the floxed alleles results a deletion mutant transcript [31]. We first verified that Cre-based recombination occurs in keratinocytes, we isolated mouse keratinocytes from C57.*Pparg*-/-^epi^ mice and wildtype (WT) sibling controls and performed qRT-PCR to verify the presence of deletional mutants. Figure S1 shows that wildtype keratinocytes express only the 700 bp wildtype allele. In contrast, the 300 bp mutant transcript was detected only in keratinocytes obtained from C57.*Pparg*-/-^epi^ mice. The presence of wildtype *Pparg* transcripts in the C57.*Pparg*-/-^epi^ mice is likely reflective of fibroblast contamination.

**Figure 1:**
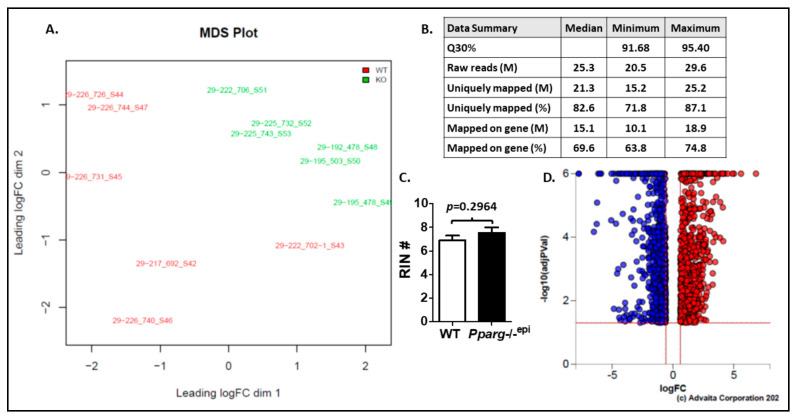
Multi-dimensional scaling (MDS) and quality control (QC) data summary from RNA sequencing data and changes in the transcriptome seen in C57.*Pparg*-/-^epi^ mice. (**A**) Multi-dimensional scaling plot provides a visual presentation of the similarities/distances among the experimental samples. Distances on the plot correspond to the leading fold-change between each pair of samples. The leading fold change is calculated as the average (root-mean-square) of the largest absolute log-fold changes between each pair of samples. Ideally, samples would cluster well within the main condition/treatment of interest. (**B**) No significant difference is seen in starting RNA quality. RNA sequencing data and QC summary. (**C**) The mean and SEM for the RNA integrity number (RIN) for the WT and C57.*Pparg*-/-^epi^ (*Pparg*-/-^epi^) samples are shown. Range = 5.9 to 8.6 and 5.7 to 8.6 for WT and *Pparg*-/-^epi^ mice, respectively. Two-tailed *t*-test. (**D**) Volcano plot of differentially expressed genes (Advaita Corp, Ann Arbor, MI). All significant differentially expressed genes are represented in terms of their measured expression change (*x*-axis; log2(FC) and the significance of the change (*y*-axis). The significance is represented in terms of the negative log (base 10) of the FDR, so that more significant genes are plotted higher on the *y*-axis. The dotted lines represent the thresholds used to select the DE genes: │log2 fold-change│ ≥ 0.6 and < 0.05 false discovery rate (FDR). The up-regulated genes are shown in red, while the down-regulated genes are blue.

We next examined how the loss of epidermal *Pparg* altered the transcriptome in the epidermis and the superficial dermis. After the isolation of total RNA from epidermal scrapings of C57.*Pparg*-/-^epi^ (KO) mice and WT sibling controls, we performed mRNA sequencing. Multi-dimensional scaling of the mRNA sequencing data for each of the WT and C57.*Pparg*-/-^epi^ skin showed good separation and clustering of the experimental groups (Figure 1A). Figure 1B shows a summary of the mRNA sequencing data. The read quality was good, with a minimum of 91.8% of reads having a base call accuracy of 99.9% (Q30). After aligning reads to the mouse reference genome (mm10) and performing gene annotation, differential expression (DE) analysis was performed (Supplemental Table S1). In Figure 1C, we show that there was no significant difference in the RNA integrity numbers (RIN) for the WT and C57.*Pparg*-/-^epi^ mouse groups. It should be noted that RNA was extracted from non-lesional tissue in the absence of any external stimuli, thus, the DE analysis represents alterations in the normal physiologic transcriptome. Under these basal conditions, loss of epidermal *Pparg* was associated with altered expression of 1707 out of 14,482 (11.78%) transcripts (≥1.5-fold change; FDR < 0.05) (Figure 1D and Supplemental Table S1).

**Figure 2:**
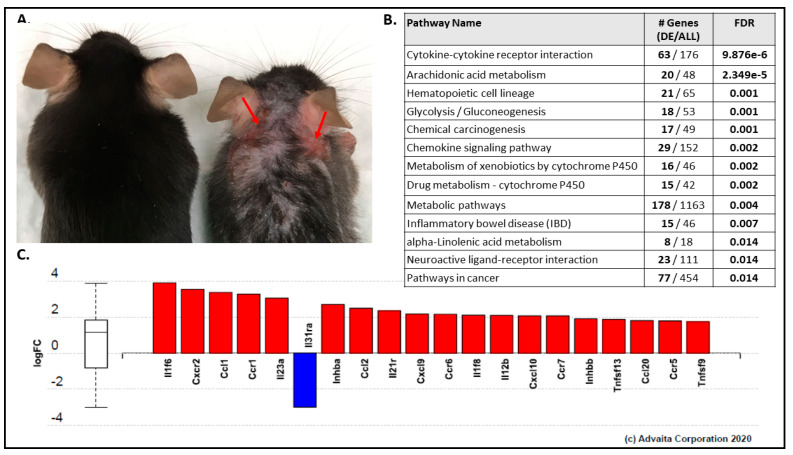
C57.*Pparg*-/-^epi^ mice exhibit alopecia and cutaneous lesions while the top KEGG pathway that maps to the differential expression analysis is associated with inflammatory signaling. (**A**) Representative photograph of a C57.*Pparg*-/-^epi^ mouse (right) and a wildtype littermate (left). Areas of erythematous skin lesions that develop spontaneously in these mice are noted by the red arrows. Alopecia and skin flaking are also observed. (**B**) Differentially expressed genes were mapped to KEGG pathways using iPathwayGuide (Advaita Corporation. Thirteen KEGG pathways mapped to our data set with an FDR < 0.05. (**C**) The top 20 of 63 genes that mapped to the cytokine-cytokine receptor interaction KEGG pathway (iPathwayGuide, Advaita, KEGG:04060) are shown. Gene mapping was restricted to genes that had a │log2 FC│ ≥ 0.6 at an FDR < 0.05. Up-regulated genes are shown in red columns and down-regulated genes are shown with blue columns. The *Y*-axis represents the log2 fold change (logFC).

Phenotypically, loss of epidermal *Pparg* was also associated with an altered skin phenotype in nearly all mice (Figure 2A and Figure S2, Supplemental Table S2). Partial alopecia and dry flaky skin with dandruff was seen in most C57.*Pparg*-/-^epi^ mice. Inflamed skin lesions, either seen as blepharitis or visible erythematous patches, were also highly prevalent (Supplemental Table S2). As mice aged, they exhibited increased numbers of visible wounds, with 75% of C57.*Pparg*-/-^epi^ mice at 18–23 weeks exhibiting wound lesions (Supplemental Table S2). The skin lesions were seen to be most prevalent on the head, the caudal aspect of the ears, and the forward flanks of the mice. C57.*Pparg*-/-^epi^ mice also exhibited increased scratching behavior, which was also observed in mice with a cytokeratin-15-driven loss of *Pparg* [13]. Thus, the wounded areas were likely due to self-mutilation. 

Using iPathwayGuide (Advaita Bioinformatics, Ann Arbor, MI, USA), we next performed pathway analysis by comparing the DE data set against the Kyoto Encyclopedia of Genes and Genomes (KEGG) database. Thirteen different KEGG pathways were matched to the DE analysis with an FDR cutoff <0.05 (Figure 2B). The top KEGG pathway was the cytokine–cytokine receptor interaction (KEGG:04060), in which 63 of 176 genes in the KEGG gene set were significantly altered in the DE analysis. Figure 2C shows the top 20 genes that annotate to this KEGG pathway (see Supplemental Table S3 for all 63 genes). Of the 63 gene transcripts, 44 were upregulated (69.8%).

Gene ontology (GO) analysis also indicated a role for epidermal *Pparg* in regulating inflammation. Molecular function GO term analysis returned cytokine activity (GO:0005125) as the top correlated GO term (Figure S3A; see Supplemental Table S4 for all 42 genes in the DE data set that correlate with the cytokine activity GO panel). As seen in Figure 2C and Figure S3B, with the exception of one transcript (*Il31ra*), all of the top regulated pro-inflammatory transcripts were increased by loss of epidermal *Pparg*. This supports previous reports that PPARγ agonists have anti-inflammatory activity in skin [5,18,19,20,21,22,23,24,25,26]. It also indicates that epidermal PPARγ serves as a key modulator of baseline cutaneous inflammatory signaling.

**Figure 3:**
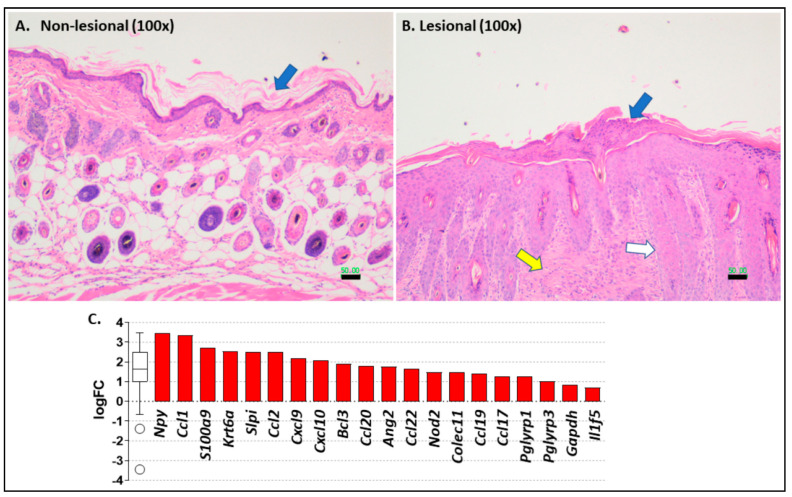
The cutaneous lesions in C57.*Pparg*-/-^epi^ mice exhibit features consistent with an inflammatory dermatosis. (**A**) Representative photomicrograph of a hematoxylin & eosin (H&E) stain slide of non-lesional skin from a C57.*Pparg*-/-^epi^ mouse. While hyperkeratosis was observed (blue arrow in **A**), the epidermis did not exhibit evidence of hyperplasia, and no significant inflammatory infiltrate was observed. Sebaceous glands are also absent.Scale bar = 50 µm. (**B**) Representative H&E of lesional skin from a C57.*Pparg*-/-^epi^ mouse. The lesions show areas of parakeratosis and intracorneal serum, neutrophils, and focal hemorrhage (blue arrow in **B**). The epidermis was acanthotic and mildly spongiotic with subjacent proliferation of hair follicle epithelium (white arrow). Lesional skin was also associated with increased inflammatory infiltrates and areas of dermal fibrosis (yellow arrow). Scale bar = 50 µm. (**C**) The top 20 of 23 genes that mapped to the antimicrobial humoral response GO term are shown (iPathwayGuide, Advaita, GO:0019730; *p*-value = 1.64E-04). Gene mapping was restricted to genes that had a │log2 FC│ > 0.6 at an FDR < 0.05. The *Y*-axis represents the log2 fold change (logFC). Up-regulated genes are shown in red columns. The box represents the 1^st^ quartile, the median and the 3^rd^ quartile for all genes in the GO term. The whiskers represent the minimum and maximum, and the circles represent the outliers.

In Figure 3, we show representative photomicrographs of non-lesional (Figure 3A) and erythematous lesional skin (Figure 3B) in C57.*Pparg*-/-^epi^ mice. Non-lesional skin showed hyperkeratosis and the absence of visible sebaceous glands (discussed below). Lesional skin shows areas of parakeratosis and intracorneal serum, neutrophils, and focal hemorrhage (Figure 3B). C57.*Pparg*-/-^epi^ lesional epidermis was acanthotic and mildly spongiotic with subjacent proliferation of hair follicle epithelium. Lesional skin was also associated with increased inflammatory infiltrates and areas of dermal fibrosis.

Upon injury or pathogen exposure, damage-associated molecular patterns (DAMPs) released by damaged cells or pathogen-associated molecular patterns (PAMPs) are detected by pattern recognition receptors (PRR) and upregulate the expression of alarmins that have direct antimicrobial activity or stimulate the innate immune system [32]. Given that the skin is a major barrier to environmental pathogens, it is interesting that loss of epidermal *Pparg* also strongly correlated with antimicrobial pathways (Mus musculus GO: 0019730, Figure 3C and Supplemental Table S5). This panel includes a number of known alarmins and/or antimicrobial peptides: *S100a9* [33], *Krt6a* [32], *Slpi* [34], *Colec11* [35,36], and *Pglyrp1* and *3* [37]. The top up-regulated gene transcript, neuropeptide Y (*Npy*), has also been shown to regulate inflammation and to promote the release of the alarmin HMGB1 (high mobility group box 1) from mouse macrophages [38]. While not a member of this GO panel, it should be noted that *S100a8* is also a known alarmin [33] and was also strongly elevated in C57.*Pparg*-/-^epi^ mouse skin (6.65-fold, FDR = 1.16E-07). 

*Ccl1, Ccl2*, Ccl17, Ccl19, *Cxcl9*, and *Cxcl10* all represent kinocidin chemokines that contain a γ-core motif characteristic of classical AMPs and have direct microbicidal activity [39]. *Cxcl12* (2.25-fold; Supplemental Table S1), and *Ccl7* (2.83-fold; Supplemental Table S4) represent additional chemokines that contain the γ-core domains [39], yet are not included in the GO: 0019730 panel. CCL20 is also known to have potent antimicrobial activity due to structural features similar to human β-defensin-2 [40]. The increase in the B-cell leukemia 3 gene (*Bcl3*) is also interesting as this gene product is a member of the IκB protein family and acts to promote the expression of alarmin and AMP genes in keratinocytes [33]. Angiopoietin-2 (*Ang2*) promotes angiogenesis and is shown to promote survival in mouse sepsis models [41]. An additional AMP that was not listed in the GO panel in Figure 3C and Supplemental Table S5 is defensin beta 6 (*Defb6*) (Supplemental Table S1). Defensin beta 6 (5.71-fold change, FDR = 1.70E-04) is a member of the class of beta defensins that play a key role in epidermal barrier protection as broad-spectrum microbicidal agents with additional immunomodulatory activity [42]. 

### 2.2. Differential Expression Analysis Indicates That C57.Pparg-/-^epi^ Mice Exhibit Increased Expression of Inflammasome Mediators

From the above data, it is clear that the loss of epidermal *Pparg* promotes a pro-inflammatory phenotype. Alarmins such as the calgranulins (*S100a8* and *9*) trigger inflammation by promoting inflammasome activation and downstream pyroptosis. Inflammasome-mediated pyroptosis is a form of pro-inflammatory programmed cell death in which cellular contents are released through pores formed by proteins known as gasdermins [43,44]. GO analysis of biological processes also linked to genes annotated to pyroptosis. A total of 9/17 genes in the Mus musculus pyroptosis GO term (GO:0070269; *p*-value = 3.30E-04) that were identified in our sequencing data were significantly altered (log2FC > 0.6; FDR < 0.05) (see Supplemental Table S6). We found that gasdermin A2 was increased over 17-fold in the skin of C57.*Pparg*-/-^epi^ mice (Table 1 and Supplemental Table S6). We therefore examined the genes in the pyroptosis GO panel along with other mediators of the inflammasome and pyroptosis pathway (Table 1). In the canonical NOD-like receptor-mediated pyroptosis pathway in humans, inactive inflammatory caspase-1 monomers are recruited to the activated inflammasome sensor proteins to form an active complex that in turn cleaves gasdermin D to initiate active pore-forming ability [43,44]. Activated pro-inflammatory caspases also act to cleave IL1 family cytokines such as IL1β and IL18 to their active forms, initiating potent inflammatory signaling.

Pyroptosis is initiated by pathogens and host-derived damage or inflammatory signals that activate inflammasome sensors, including the family of nucleotide-binding oligomerization domain (NOD)-like receptors (NLR) and absent in melanoma 2 (AIM2)-like receptors [45]. In addition to the increase in gasdermins, inflammasome sensor protein transcripts Aim2, *Nod2* and *Nlrp3* were also elevated in C57.*Pparg*-/-^epi^ skin. *Aim2* (3.67-fold increase) is known to form inflammasome complexes in response to cytoplasmic dsDNA in inflammatory skin disease [46]. In addition to an increase in the expression of the inflammasome sensors and gasdermins, C57.*Pparg*-/-^epi^ mice also had an associated increase in transcripts for the inflammatory caspases, *Casp4* and *Casp1* (Table 1).

The IL1 family is composed of seven pro-inflammatory family members ((*Il1a* (IL-1α), *Il1b* (IL-1β), *Il18* (IL-18), *Il33* (IL-33), *Il1f6* (IL-36α), *Il1f8* (IL36β), and Il1f9 (IL36γ)), and four anti-inflammatory members (*Il1f7* (IL-37), *Il1f10* (IL-38), *Il1rn* (IL1R antagonist), and *Il1f5* (IL36R antagonist)) [48]. Of the pro-inflammatory family members, all but *Il1a* and *Il33* were significantly upregulated in C57.*Pparg*-/-^epi^ mice (Table 1 and Supplemental Table S1). In contrast, of those IL1 family members with antagonistic activity, only *1l1f5* (IL36RN) was significantly elevated (Table 1), while *Il1rn* and *Il1f10* (IL38) were not significantly altered and IL37 (*Il1f7*) was not identified in the mRNA seq data set (Supplemental Table S1). In addition, other inhibitors of inflammasome-mediated inflammation (*Il18bp* and *Nlrp10* [45,49,50]) were not significantly altered by loss of epidermal *Pparg* (FDR >0.05) (Supplemental Table S1). It might also be noted that *Il1f5* acts to antagonize the effects of *Il1f6, Il1f8*, and *Il1f9* [49,50]. Thus, if the relative levels of *Il1f6, Il1f8*, and *Il1f9* (14.88-, 4.30-, and 1.85-fold, respectively) and *Il1f5* (1.63-fold) are reflective of their respective protein expression and activities, then IL-36α, β and γ could be important contributors to the observed inflammatory phenotype observed in our C57.*Pparg*-/-^epi^ mice.

### 2.3. Skin Lesions in C57.Pparg-/-^epi^ Mice Show Elevation of Cytokines Associated with Inflammatory Dermatoses 

**Figure 4:**
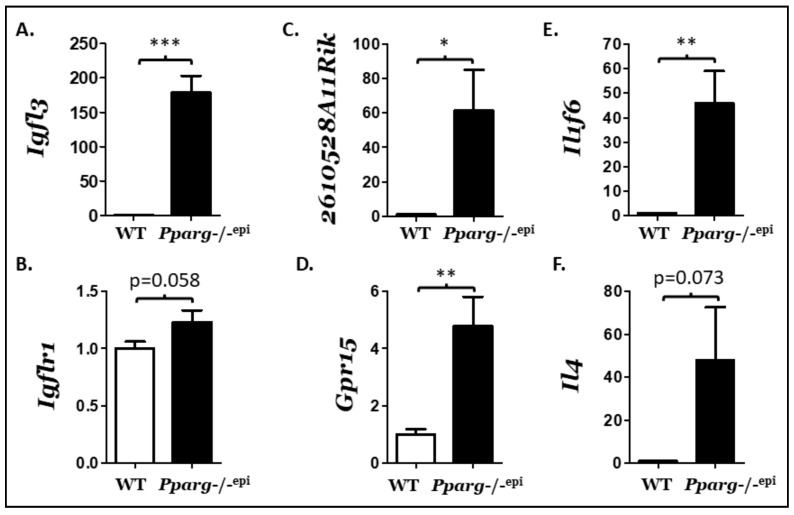
Confirmation of transcriptional upregulation for mediators associated with inflammatory skin disease. Total RNA was extracted from full-thickness non-lesional skin of C57.*Pparg*-/-^epi^ (*Pparg*-/-^epi^) mice and wildtype (WT) sibling controls. Quantitative RT-PCR was then performed, and results were normalized to U. Results represent the fold-change in individual transcript levels between *Pparg*-/-^epi^ and WT controls. (**A**) Relative expression of *Igfl3*. (**B**) Relative expression of the IGFL3 receptor, *Igflr1*. (**C**) Relative expression of 26*10528A11Rik*. (**D**) Relative expression of the 2610528A11Rik receptor, *Gpr15*. (**E**) Relative expression of *Il4*. (**F**) Relative expression of *Il1f6* (IL36α). *, *p* < 0.05; **, *p* < 0.01; ***, *p* < 0.001, 2-tailed *t*-test with Welch’s correction.

As seen in Figure 2 and Figure 3 and Table 1, many well-characterized inflammatory mediators were altered with loss of epidermal *Pparg*. In addition, we also found two highly upregulated transcripts that are associated with inflammatory skin disease, but for which there is limited knowledge regarding their respective mechanisms of action. The first of these, the IGF-like family member 3 (*Igfl3;* FC = 48.30, FDR = 9.52E-13), represents the second most up-regulated gene transcript in the differential expression analysis. *Igfl3* is the mouse ortholog of the human *IGFL1* gene [51]. He expression of *Igfl3* or the human *IGFL1* is primarily localized to skin, with increased expression occurring following wounding [51], psoriatic-like inflammation [51] and atopic skin disease [52,53]. It is known to be induced by TNFα, but not other psoriasis-associated cytokines, and has been shown to bind with high affinity to the tumor necrosis factor receptor family member TMEM149 (renamed *Igflr1*) [51]. *Igflr1* is known to be expressed on mouse T cells [51]. In Figure 4A,B, we examined the expression of *Igfl3* and its receptor *Igflr1* by qRT-PCR using RNA extracted from whole skin rather than epidermal scrapings. Whole skin was used in this case as *Igflr1* and *Gpr15* are expressed in immune cells that might not be present at high levels in the epidermis or superficial dermis. *Igfl3* was elevated 178.9-fold in full thickness C57.*Pparg*-/-^epi^ skin relative to wildtype skin (Figure 4A). In contrast, there was no significant change in the expression of its known receptor, *Igflr1* (Figure 4B). 2610528A11Rik is the second poorly characterized gene product that was also highly elevated (14.84-fold) (see Figure S3 and Supplemental Table S4). Given that a number of antimicrobial peptides were seen to be elevated in C57.*Pparg*-/-^epi^ skin, it is interesting that the human ortholog of 10528A11Rik, C10orf99, is a CC-motif chemokine that was renamed AP-57 (antimicrobial peptide with 57 amino acids) after it was found to have antimicrobial activity [54]. Moreover, 2610528A11Rik is expressed heavily in tissues exposed to environmental pathogens, including the colon, esophagus, and skin [54,55]. Mice lacking 2610528A11Rik are found to exhibit an increased ratio of CD4/CD8 cells and decreased IgM level, suggesting that this poorly characterized chemokine may play a role in immune regulation [55]. As with *Igfl3*, increased expression of *2610528A11Rik* and its human ortholog are linked to mouse models of skin wounding and psoriasis [51], human psoriasis [51,56], and human atopic dermatitis [52,53]. In Figure 4C, *2610528A11Rik* transcripts was elevated 61.53-fold by quantitative RT-PCR from RNA isolated from full-thickness non-lesional C57.*Pparg*-/-^epi^ mouse skin relative to wildtype control mice.

Recently, it was found that 2610528A11Rik is a high-affinity ligand for the T-cell chemoattractant G-protein coupled receptor GPR15 [57,58]. GPR15 is expressed on effector and regulatory T-cells, precursor dendritic epidermal T-cells (TCRVγ3^+^TCRγ/δ^+^ T-cells), memory B-cells, and plasmablasts [57,58]. Consistent with a possible role for 2610528A11Rik in mediating GPR15^+^ cell recruitment, *Gpr15* transcripts were significantly elevated 4.79-fold in C57.*Pparg*-/-^epi^ skin relative to WT skin.

In Figure 2C, Figure S3, and Table 1, the top up-regulated cytokine transcript in C57.*Pparg*-/-^epi^ mouse skin was *Il1f6* (IL36α) (14.88-fold). As with *Igfl3* and *2610528A11Rik*, studies suggest that *Il1f6 (*IL36α) plays an important role in both psoriasis and atopic dermatitis [39,50,59]. The overexpression of *Il1f6* in a transgenic mouse results in a phenotype similar to that observed in our study and is characterized by acanthosis, hyperkeratosis, inflammatory infiltrates, and increased cytokine and chemokine expression [49]. Moreover, loss of *Il1f6* is capable of blocking the skin inflammation seen in an imiquimod-induced psoriatic mouse model [50]. We therefore assessed the expression of the *Il1f6* gene by qRT-PCR using RNA extracted from whole skin. As seen if Figure 4E, *Il1f6* transcripts were elevated 45.77-fold in C57.*Pparg*-/-^epi^ non-lesional skin relative to WT sibling controls.

The above data indicate that C57.*Pparg*-/-^epi^ mouse develop spontaneous inflammatory skin lesions and express inflammatory mediators associated with inflammatory dermatoses. Atopic dermatitis is associated with T-cells exhibiting a Th2 polarized phenotype, with increased expression of Th2 cytokines, such as IL4, Il5, and IL13 [60]. In contrast, psoriasis is associated with Th17 T-cells producing Th17 cytokines such as IL17A and IL22 [61,62]. However, our DE expression analysis failed to detect any of these cytokine transcripts. One possible explanation was that the epidermal scrapings were not deep enough to capture sufficient numbers of dermal T-cells. We therefore assessed *Il4*, *Il17* and *Il22* expression by qRT-PCR of RNA obtained from the full-thickness skin. Again, we failed to detect *Il17* and *Il22* transcripts. However, low levels of Il4 transcripts were detected, with a non-significant increase in *Il4* transcripts observed in C57.*Pparg*-/-^epi^ skin (Figure 4F).

### 2.4. Pparg-/-^epi^ Mice Are Characterized by a Marked Loss of Fatty Acid Metabolic Enzymes and an Asebia Phenotype

**Figure 5:**
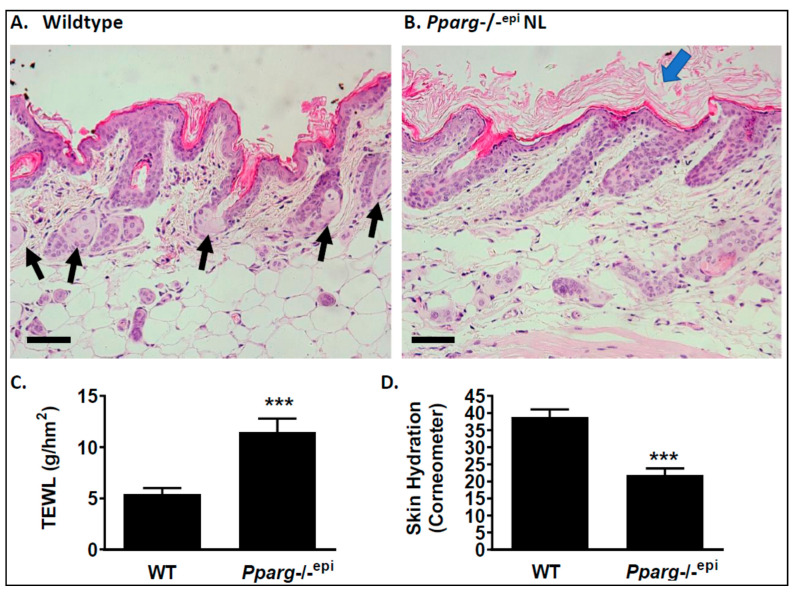
Loss of epidermal Pparg results in an asebia phenotype with hyperkeratosis. (**A**) Representative photomicrograph of a hematoxylin & eosin (H&E) stained slide of skin from a sibling wildtype control mouse. Sebaceous glands are noted by the black arrows. Scale bar = 50 µm. (**B**) Representative photomicrograph of a H&E-stained slide of skin from a *Pparg*-/-^epi^ mouse (SKH1 background). As in C57.*Pparg*-/-^epi^ mouse epidermis (Figure 3A), hyperkeratosis was observed (blue arrow). However, the absence of visible sebaceous glands was again apparent (see also Figure 3A). Scale bar = 50 µm. (**C**) Transepidermal water loss (TEWL) is increased in *Pparg*-/-^epi^ relative to wildtype sibling controls. (**D**) Skin hydration is reduced in *Pparg*-/-^epi^ relative to wildtype sibling controls. ***, *p* < 0.001, 2-tailed *t*-test.

In Figure 5A, we demonstrate that the loss of epidermal *Pparg* results in an asebia phenotype. In Figure 5A, sebaceous glands are clearly seen in wildtype SKH-1 mouse skin. In contrast, a complete loss of visible sebaceous glands and a thickened stratum corneum are seen in non-lesional *Pparg*-/-^epi^ mouse skin (SKH-1 background) (Figure 5B). A similar loss of sebaceous glands and hyperkeratosis was noted in C57.*Pparg*-/-^epi^ (C57BL/6 background) (Figure 3A). In Figure 5C, *Pparg*-/-^epi^ mice also exhibited a significant increase in transepidermal water loss (TEWL). This increase in water loss in *Pparg*-/-^epi^ mice was accompanied by a decrease in skin hydration (Figure 5D). Grossly, these areas of non-lesional C57.*Pparg*-/-^epi^ skin are characterized by hyperkeratosis or a marked increase in the stratum corneum (blue arrows in Figure 3B and Figure 5B) that is consistent with the grossly visible dandruff seen in Figure S1.

In Table 2, we show that transcripts associated with cutaneous sebum production are markedly reduced: fatty acid 2-hydroxylase (*Fa2h*), stearoyl-coenzyme A desaturase (*Scd3*), acyl-CoA wax alcohol acyltransferase 1 (*Awat1*) and fatty acyl CoA reductase 2 (*Far2*) represented the first, second, fourth, and seventh most reduced transcripts seen in C57.*Pparg*-/-^epi^ mouse skin, respectively. FA2H in mouse skin is restricted to the sebaceous glands, where it synthesizes 2-hydroxylated glucosyl-ceramide [63]. Both FA2H and FAR2 also play a role in wax ester synthesis in sebaceous glands [63].

In addition, genes associated with lipid metabolism and cutaneous lipid barrier function were significantly altered by the loss of epidermal *Pparg*. *Acoxl* encodes an acyl-coenzyme A oxidase-like peroxisomal protein for which very little is known, although it belongs to a family of enzymes that are necessary for peroxisomal alpha- and beta-oxidation [69,70]. The chromosomal region where *ACOXL* resides (2q13) was also mapped as a possible susceptibility locus in human alopecia areata [69]. Solute carrier family 27, member 2 (*Slc27a2*), or very-long-chain acyl-CoA synthetase converts long-chain, branched-chain and very-long-chain fatty acids containing 22 or more carbons to Acyl-CoA esters [71]. Members of the elongation of very-long-chain fatty acids-like group of enzymes (*Elovl3, 5,* and *6*) are necessary for very long chain fatty acid production that is necessary for the formation of a lipid permeability barrier [73,74]. Loss of the Δ9- desaturase function of stearoyl-coenzyme A desaturase 1 (*Scd1*) results in a skin phenotype similar to that observed in our mouse model [14]. This includes hyperkeratosis, transepidermal water loss and xerosis, sebocyte hypoplasia, and increased inflammation [14]. Mitochondrial membranes require phosphatidylcholine as a major phospholipid and a reduction of phosphatidylcholine transfer protein (*Pctp*) in mice results in mitochondrial dysfunction, oxidative stress, and altered barrier function in lung [72]. Carnitine acyltransferase (*Crat*) is a peroxisomal enzyme that catalyzes the transfer of acyl groups from carnitine to coenzyme A and is important in the transport of fatty acids for beta-oxidation [75].

The above data are all indicative of a severe defect in the formation of the lipid epidermal water barrier in mice lacking epidermal *Pparg*. We therefore examined the epidermal and sebaceous gland lipid content using Oil Red O staining (Figure S4). While lipid-laden sebocytes were seen in holocrine sebaceous glands in wildtype mice (Figure S4A), these lipid-laden glandular cells were absent in C57.*Pparg*-/-^epi^ mice (Figure S4B). In hair follicles, lipid staining was limited to small deposits within the hair follicle sheath. In addition, wildtype mice showed lipid staining within the stratum corneum. However, lipid staining was largely absent in the thickened stratum corneum of C57.*Pparg*-/-^epi^ mice (Figure S4B).

### 2.5. The Hyperkeratosis and Permeability Barrier Disruption Seen in Pparg-/-^epi^ Mice Correlates with Transcriptomic Changes Linked to Increased Cornification and the Epidermal Differentiation Complex (EDC)

Given the hyperkeratosis observed in mice lacking epidermal *Pparg*, it is not surprising that genes associated with keratinocyte terminal differentiation and corneocyte formation are increased in C57*.Pparg*-/-^epi^ mouse skin (Table 3). GO terms annotated to peptide cross-linking and keratinization were found to be associated with the changes in the transcriptome of C57.*Pparg*-/-^epi^ mice (Table 3). Four of the top six keratins (*Krt16, Krt17*, and *Krt6a* and *b*) that were increased in mice lacking epidermal *Pparg* are keratins that are known to serve as alarmins and are up-regulated in wound healing and psoriasis [32,76]. The upregulation of these keratins promotes epidermal hyperplasia and activation of the innate immune system [32]. It has been shown that the upregulation of these hyperplastic and pro-inflammatory keratins may be linked to inflammasome activation following skin injury [32]. In contrast, basal cell layer keratins (*Krt14* and *Krt5*) and suprabasal keratins (*Krt1* and *Krt10*) were not significantly altered. Interestingly, *Krt15* is specific to hair bulb stem cells [77]. Since *Krt15* transcription is known to be suppressed in hyperproliferative skin and in wound healing [30,76], the decrease in *Krt15* transcripts is not surprising. However, given the observed alopecia, it is somewhat surprising that transcripts for hair-follicle-specific keratins (*Krt6a, Krt6b*, and *Krt17*) were increased. Other hair-follicle- (e.g., *Krt25–28, Krt71–74,* and *Krt 75*) and hair-specific keratins (e.g., *Krt31–40*, and *Krt81–86*) were not significantly altered (FDR > 0.05) (Supplemental Table S1).

In addition to the increase in the wound response keratins, there is a marked upregulation of genes that are members of the EDC. The EDC is a large gene cluster complex located at 1q21 in humans (3q in mice) [78]. This gene complex contains four gene clusters coding for structural proteins of the cross-linked envelope of corneocytes. This includes filaggrin (*Flg*)-like (e.g., *Inv, Hrnr*), late cornified envelope (*Lce*), and small proline rich region (*Sprr*) proteins, as well as a cluster containing the alarmin S100 genes [78]. The cross-linking of these proteins into the cornified envelope is in turn dependent on the expression and activation of epidermal-specific caspase 14 and transglutaminase such as transglutaminase 3 [79]. Reduced expression of components of the cornified envelope, loricrin, filaggrin and involucrin are frequently down-regulated in human atopic dermatitis [80,81]. However, involucrin (*Ivl*) was up-regulated in C57.*Pparg*-/-^epi^ skin (Table 3), while filaggrin family member 2 (*Flg2*) and loricrin (*Lor*) were not significantly altered (FC = −1.10, −1.05, respectively (FDR >0.05) (Supplemental Table S1).

## 3. Discussion

One of the surprising findings of this study was the degree to which the cutaneous transcriptome is altered by loss of epidermal *Pparg*. Nearly 12% of transcripts were significantly altered in C57.*Pparg*-/-^epi^ epidermal scrapings relative to wildtype mice. This degree of disruption to the cutaneous transcriptome cannot be adequately addressed in a single manuscript. However, it is clear that epidermal PPARγ plays a key role in regulating normal homeostatic functions of the skin. In particular, the changes in the transcriptome and the observed spontaneous inflammatory lesions indicate that PPARγ acts as a major modulating influence on cutaneous inflammatory signaling. In addition, similar to a previous report using a *Krt15*-promoter-driven loss of *Pparg* [13], we show that *Krt14*-promoter-driven loss of *Pparg* is associated with an asebia phenotype. The loss of visible sebaceous glands is accompanied by loss of transcripts that encode sebaceous gland markers and enzymes associated with sebum production. The asebia phenotype was also accompanied by partial alopecia, hyperkeratosis, and a loss of barrier function that resulted in increased water loss and xerosis.

The important role of epidermal PPARγ in moderating cutaneous inflammation is seen both by analysis of the differentially expressed genes as well as the observed phenotype. The top GO terms that linked to our data set showed upregulation of multiple inflammatory cytokine and chemokine transcripts, upregulation of genes associated with inflammasome activation (*Aim2*, *Nod2*, *Nlrp3*, *Il1f6* and *8*, *Casp1*, *Gsdma2*, *Gsdmc*), and the upregulation of keratins associated with inflammatory hyperplasia (e.g., *Krt 6, 16, 17*). In addition to the disruption in inflammatory signaling, C57.*Pparg*-/-^epi^ mice developed spontaneous lesions that are reminiscent of wound healing, atopic dermatitis, and psoriasis. While it is unclear whether the inflammatory lesions simply represent reactive wound healing responses to aggressive scratching behavior, there is evidence that these lesions may represent a model of inflammatory skin disease. Both atopic dermatitis and psoriasis are associated with localized and generalized pruritus, and psoriasis can appear in areas of traumatized skin (Koebner phenomenon). The presence of low levels of Il4 but no detectable Il17 or IL22 suggest that the lesions in our mice may represent a model of atopic dermatitis. However, the IL4 expression was very low and the absence of IL17 and IL22 could simply reflect less efficient RT-PCR primers. Moreover, a major limitation of our study is that the data that we obtained was from non-lesional skin. Thus, future work will focus on examining differences in lesional versus non-lesional skin and performing immunophenotyping of infiltrating inflammatory cell populations.

In addition to the inflammatory lesions, C57.*Pparg*-/-^epi^ mice exhibited an asebia phenotype with hyperkeratotic skin, alopecia, and markedly reduced lipid content in the epidermal stratum corneum. The absence of visible sebaceous glands following loss of *Pparg* expression in the basal layer of the epidermis is interesting as this model resembles mice with a homozygous mutation of the coenzyme A desaturase-1 (*Scd1*) gene [82]. These mice also exhibit alopecia and dry flaky skin and some features consistent with psoriasis, although these mice lack the T-cell infiltration characteristic of psoriasis [82]. While *Scd1* mRNA was modestly reduced in our mouse model, *Scd3* mRNA expression was suppressed > 200-fold. The asebia phenotype was also associated with a marked decrease in the *Elovl3* gene product, which is restricted to the sebaceous glands and epithelial cells of the hair follicle in mice [67]. As seen in Table 2, transcripts for numerous genes associated with very-long-chain fatty acid and wax ester synthesis are decreased. Thus, the absence of visible sebaceous glands may simply represent the inability to form the lipid constituents of sebum. Nonetheless, the observed hyperkeratosis, increased transepidermal water loss and dry skin are all expected outcomes from a loss of the lipid permeability barrier.

Our mouse model retains some of the features, but also exhibits differences to mice with targeted deletion of *Pparg* in Krt15^+^ bulb stem cells. Loss of *Pparg* within follicular bulb stem cells results in a progressive scarring alopecia such as that observed in the human disease Lichen planopilaris (LPP) [13]. These follicle-specific *Pparg* knockout mice were born with normal hair and skin [13]. However, the mice developed lymphocyte-rich inflammatory lesions of the follicular pilosebaceous units that began to appear approximately 3 months after birth [13]. The inflammatory lesions led to disruption of the pilosebaceous units, resulting in a delayed alopecia and hypoplastic sebaceous glands, as well as dry hyperkeratotic skin and increased scratching behavior [13]. In contrast, in our *Krt14*-Cre-driven knockout model, we observed the absence of visible sebaceous glands in the skin of pups shortly after birth (not shown). We also did not observe the presence of perifollicular lymphocyte-rich infiltrates and the dermal scarring that was associated with the alopecia observed in the *Krt15-*Cre-driven *Pparg* knockout mice.

Microarray analysis of inflamed mouse follicular tissue following *Krt15* promoter-driven deletion of *Pparg* showed increases in multiple cytokines, tissue remodeling enzymes, apoptosis regulators, and eicosanoid signaling molecules [13]. As we also found an increase in multiple pro-inflammatory genes, it is surprising that with the exception of *Ccl19*, which was modestly elevated in the C57.*Pparg*-/-^epi^ mouse skin, the other up-regulated genes associated with the *Krt15*-Cre-driven *Pparg* knockout mice were not significantly altered (<│1.5│-fold change or > 0.05 FDR) in our mouse model. Similarly, as in our mouse model, *Krt15*-Cre-driven loss of *Pparg* resulted in the down-regulation of multiple genes associated with fatty acid metabolism, cholesterol biosynthesis, fatty acid metabolism, peroxisome biogenesis, and hair follicle keratins [13]. However, there was limited overlap, as only 9 of 49 genes associated with these processes in the *Krt15*-Cre mouse model were also down-regulated in our *Krt14*-Cre model (*Acaa1b, Soat1, Scd1, Elovl3, Elovl5, Elovl16, Mgll, Krt6a*, and *Krt15*). The differences in the phenotype and transcriptomic changes may be due to the fact that in our model, deletion of *Pparg* occurs throughout the basal layer of the epidermis, not the follicular stem cell population. In addition, unlike our study, the prior microarray studies that used *Krt15*-Cre-driven loss of *Pparg* were focused on skin areas with perifollicular inflammation.

In conclusion, we show that the loss of epidermal *Pparg* is associated with a dramatic change in the transcriptome. The large number of alterations to transcripts encoding inflammatory mediators, the inflammasome, keratinocyte differentiation and keratinization, and lipid barrier synthesis are somewhat surprising given that the mice were not exposed to any external stimuli or stress. The transcriptomic changes are also accompanied by an asebia phenotype, partial alopecia, a defect in barrier function, evidence of increased keratinization, and an increase in DAMPs. The complex phenotype seen in these mice makes it difficult to determine which of the observed phenotypic and transcriptomic changes are directly attributable to the transcriptional activity of PPARγ. Given that PPARγ is known to directly regulate the transcription of adipogenic genes, it is possible that the loss of epidermal *Pparg* has a direct effect on the lipid barrier. Since defects in permeability function are associated with an increase in exposure to environmental stimuli, it is not surprising that this could then lead to an increase in inflammatory mediator release and hyperkeratosis. Alternatively, PPARγ is known to suppress inflammatory signaling through transrepressive mechanisms [1]. Thus, loss of epidermal *Pparg* could directly promote increased inflammatory mediator release through loss of its transrepressive activity. Additional studies are needed to determine the mechanisms through which epidermal *Pparg* modulates cutaneous function.

## 4. Materials and Methods

### 4.1. Animal Studies

As previously noted, mice lacking epidermal *Pparg* in the C57BL/6 background (C57.*Pparg*-/-^epi^) were obtained by crossing mice containing the floxed *Pparg* allele in the C57BL/6 background (B6.129-*Pparg^tm2Rev^*/J) with mice expressing Cre recombinase under control of the keratin 14 promoter (B6N.Cg-Tg(KRT14-cre)1Amc/J) [27]. Likewise, *Pparg*-/-^epi^ mice that were backcrossed into the SKH-1 hairless albino (SKH1-Hr^hr^) background for photocarcinogenesis studies was performed as described [28]. Mice were housed under specific pathogen-free conditions at the Indiana University School of Medicine. Mice utilized for experimental studies were between 7 and 10 weeks of age. Both male and female mice were utilized.

### 4.2. Transepidermal Water Loss and Skin Hydration

TEWL and skin hydration were measured using a Tewameter^®^ (Courage & Khazaka, Cologne, Germany) and a Corneometer^®^ (Courage & Khazaka, Cologne, Germany), as previously described [28].

### 4.3. qRT-PCR to Verify Cre-Mediated Recombination

B6.129-*Pparg^tm2Rev^*/J mice have two loxP sites flanking exons 1 and 2 of the mouse *Pparg* gene [31]. Two C57.*Pparg*-/-^epi^ mice were euthanized along with a wildtype sibling control and the dorsal epidermis was removed. Adult primary keratinocyte were isolated from the tail skin of mice sacrificed at 6–15 weeks as previously described [83]. Isolated cells were cultured in supplemented EpiLife media (Thermo Fisher, Waltham, MA, USA) on pre-coated rat tail collagen type I (Thermo Fisher, Waltham, MA, USA) tissue culture plates. Confluent keratinocytes were induced to undergo terminal differentiation by increasing the CaCl_2_ to 0.2mM for 72 h. RNA was then isolated using the Qiazol (Qiagen, Germantown, MD, USA) reagent and RNeasy Mini column clean-up. RNA was treated with DNase I (Thermo Fisher) and cDNA synthesized using SuperScriptIII First –Strand Synthesis System for RT-PCR (Thermo Fisher, Waltham, MA, USA). RT-PCR was then performed using primers that flank the two loxP sites: sense (5′-GTCACGTTCTGACAGGACTGTGTGAC-3′) and antisense (5′-TATCACTGGAGATCTCCGCCAACAGC-3′) [31]. These primers produce amplified products that distinguish the full-length (700-bp) and recombined (300-bp) transcripts [31].

### 4.4. qRT-PCR of Whole Thickness Skin

The animals were euthanized and the back skin was shaved. The skin was cleaned with 70% ethanol and then sprayed with RNase Away Reagent (Ambion by Life Technology, Waltham, MA, USA). A piece of skin was harvested with 6 mm biopsy punch, briefly rinsed in RNase Away Reagent, and immediately frozen in liquid nitrogen. The tissue was homogenized in 500 µL of Trizol (Ambion by Life Technology, Waltham, MA, USA) using BioMasher Dispensable Micro-Tube (Research Products International, Mt Prospect, IL, USA). An additional 500 µL of Trizol and 100 µL of chloroform was then added and vigorously vortexed, incubated on ice for 15 min, and then centrifuged (15000 rpm, 30 min at 4 °C). The aqueous phase was collected and the RNA was isolated using RNeasy Plus Mini Kit (Qiagen) following the manufacturer’s instruction. For each sample, 990 ng of RNA was reverse-transcribed using M-Mul V Transcriptase (Biolabs) to synthesize cDNA in 20 µL reaction volume. The cDNA was added 40 µL of TE, and 1.5 µL of the mix was used to set up real-time PCR using *Ubc* expression as an internal control. The relative expression relative to Ubiquitin C (*Ubc*) expression was performed using the ΔΔCt method [84]. All data were normalized to a mean WT data set at 1.0.

### 4.5. RNA Extraction and RNA Sequencing

Six C57.*Pparg*-/-^epi^ mice and 6 wildtype sibling controls (Cre-/-) were euthanized and non-lesional dorsal skin was excised. After snap freezing in liquid nitrogen, the epidermis was scraped from the frozen epidermis with a curette. Scraping was performed until pink-tinged tissue was observed signifying that the tissue extracted had entered the subepidermal microvascular bed of the superficial dermis. Total RNA was extracted using QIAzol and column-purified using RNeasy (Qiagen, Germantown, MD, USA). RNA integrity numbers (RIN) were assessed using a Bioanalyzer 2100 (Agilent Technologies, Santa Clara, CA, USA). The RIN of all RNA samples was 5.7–8.6. Library construction was performed using 200 ng of total RNA with a KAPA mRNA HyperPrep Kit (KK8581, ΔKapa Biosystems, Roche Sequencing and Life Science, Wilmington, MA, USA). Paired-end sequencing (2 × 75 bp) was performed by the Center for Medical Genomics at Indiana University School of Medicine using an Illumina HiSeq 4000 (Illumina Inc., San Diego, CA, USA).

### 4.6. RNA Sequencing Quality Assessment and Differential Expression Analysis

Sequencing quality was assessed using FastQC/MultiQC (version 0.11.5). To evaluate the quality of the RNA-seq data, the number of reads that fall into different annotated regions (exonic, intronic, splicing junction, intergenic, promoter, UTR, etc.) of the reference genes were determined with bamUtils (version 0.5.9) [85]. Low-quality mapped reads (including reads mapped to multiple positions) were excluded and featureCounts (version 1.5.1) [86] was used to quantify the gene level expression. Sequencing alignment was performed using STAR (version 2.5) [87] and mapped to the mouse reference genome for genome annotation (mm10/refGene). Differential gene expression analysis was performed with edgeR (version 3.22.5) [88]. In this workflow, the statistical methodology applied uses negative binomial generalized linear models with likelihood ratio tests.

### 4.7. Pathway Analysis

Pathway analysis was performed using iPathway Guide using methodology provided by Advaita Corp. iPathwayGuide scores pathways using the impact analysis method [89,90,91]. The underlying pathway topologies, comprised of genes and their directional interactions, are obtained from the KEGG database [92,93,94].

### 4.8. Gene Ontology Analysis

Gene ontology analysis was performed using iPathway Guide using the methodology provided by Advaita Corp. Using the iPathway Guide methodology, for each gene ontology (GO) term [95,96], the number of differentially expressed (DE) genes annotated to the term is compared to the number of DE genes expected just by chance.

### 4.9. Oil Red O Staining

The dorsal epidermis was excised from non-lesional doral skin of C57.*Pparg*-/-^epi^ mice and wildtype sibling controls. The skin was the snap frozen in O.C.T. and 10 µm sections cut for Oil Red O staining. After brief fixation in 10% formalin, the sections were stained with Oil Red O using the NovaUltra Oil Red O Stain Kit (IHC World, Ellicott City, MD, USA) per the manufacturer’s instructions. Slides were counterstained with Hematoxylin QS (Vector Laboratories, Burlingame, CA, USA).

## Figures and Tables

**Table 1:** Differentially expressed genes that are associated with inflammasome activation and pyroptosis [45,47].

		Gene Information	C57.*Pparg*-/-^epi^ vs. WT	
	Gene	Gene Name	Fold Change	FDR
**Inflammasome sensor proteins and caspases**	*Aim2*	absent in melanoma 2	3.67	1.11E-18
	*Nod2*	nucleotide-binding oligomerization domain containing 2	2.79	8.76E-07
	*Nlrp3*	NLR family, pyrin domain containing 3	2.14	0.025
	*Nlrp1b*	NLR family, pyrin domain containing 1B	−2.66	1.02E-06
	*Casp4*	caspase 4	2.18	1.57E-07
	*Casp1*	caspase 1	2.15	1.09E-06
**Inflammasome, IL1 family effectors and pore forming gasdermins**	*Gsdma2*	gasdermin A2	17.13	1.16E-05
	*Gsdmc*	gasdermin C	7.11	1.95E-07
	*Gsdma3*	gasdermin A3	3.76	1.24E-01
	*Gsdmc2*	gasdermin C2	2.41	1.46E-02
	*Gsdma*	gasdermin A	2.09	2.24E-09
	*IL1f6*	interleukin 1 family, member 6 (Interleukin 36α)	14.88	8.15E-10
	*Il1f8*	interleukin 1 family, member 8 (Interleukin 36β)	4.30	5.18E-24
	*Il1b*	interleukin 1 beta	2.94	2.71E-02
	*Il18*	interleukin 18	2.01	8.71E-05
	*Il1f9*	Interleukin 1 family, member 9 (Interleukin 36γ)	1.85	6.00E-04
	*Il1f5*	interleukin 1 family, member 5 (delta) (IL36RN)	1.63	3.33E-04

**Table 2:** Differentially expressed transcripts that represent sebaceous gland signature genes required for sebum production and genes required for fatty acid metabolism that are suppressed in C57.*Pparg*-/-^epi^ mice.

	Gene Information		C57.*Pparg*-/-^epi^ vs. WT		
	Gene	Gene Name	Fold Change	FDR	Ref
**Sebaceous gland signature genes**	*Fa2h*	fatty acid 2-hydroxylase	−218.14	1.53E-08	[63]
	*Scd3*	stearoyl-coenzyme A desaturase 3	−216.42	3.00E-08	[64,65]
	*Awat1*	acyl-CoA wax alcohol acyltransferase 1	−77.97	3.84E-05	[16]
	*Far2*	fatty acyl CoA reductase 2	−65.11	3.32E-07	[16,66]
	*Acaa1b*	acetyl-Coenzyme A acyltransferase 1B	−22.84	0.001012	[16]
	*Awat2*	acyl-CoA wax alcohol acyltransferase 2	−16.99	0.000744	[16]
	*Elovl3*	elongation of very long chain fatty acids-like 3	−7.59	1.60E-02	[67,68]
	*Cers4*	ceramide synthase 4	−5.22	0.00019672	[63]
	*Pdzk1*	PDZ domain containing 1	−5.78	0.011	[16]
	*Gldc*	glycine decarboxylase	−4.79	5.83E-08	[16]
	*Scd1*	stearoyl-Coenzyme A desaturase 1	−4.18	3.11E-09	[16]
	*Soat1*	sterol O-acyltransferase 1	−3.59	8.77E-04	[16]
**Fatty acid metabolism, elongation, and transport**	*Acoxl*	acyl-Coenzyme A oxidase-like	−76.37	8.18E-06	[69,70]
	*Slc27a2*	solute carrier family 27, member 2 (very long-chain acyl-CoA synthetase) (fatty acid transport protein 2 (Fatp2)	−20.49	1.88E-11	[71]
	*Scd1*	stearoyl-Coenzyme A desaturase 1	−4.18	3.11E-09	[14,64]
	*Pctp*	phosphatidylcholine transfer protein	−5.19	0.001425211	[72]
	*Elovl5*	ELOVL family member 5, elongation of long chain fatty acids	−3.91	7.67E-4	[73,74]
	*Elovl6*	ELOVL family member 6, elongation of long chain fatty acids	−2.98	1.57E-07	[73,74]
	*Crat*	carnitine acetyltransferase	−2.13	4.77E-02	[75]

**Table 3:** Differentially expressed genes that are associated with GO groups for peptide cross-linking and keratinization.

	Gene Information		C57.*Pparg*-/-^epi^ vs. WT		
	Gene	Gene Name	Fold Change	FDR	EDC Gene (Y/N)
**Genes annotated to peptide cross-linking** **(GO:0018149) (p-value = 3.900E-8)**	*Sprr1b*	small proline-rich protein 1B (Cornifin B)	12.35	1.35E-06	Y
	*Sprr2d*	small proline-rich protein 2D	9.27	1.91E-05	Y
	*Lce3e*	late cornified envelope 3E	8.72	1.51E-05	Y
	*Sprr2h*	small proline-rich protein 2H	8.37	2.72E-05	Y
	*Lce3d*	late cornified envelope 3D	8.13	4.01E-04	Y
	*Lce3f*	late cornified envelope 3F	8.07	0.00061	Y
	*Lce1g*	late cornified envelope 1G	7.26	6.15E-09	Y
	*Sprr2i*	small proline-rich protein 2I	6.33	0.00018	Y
	*Sprr2g*	small proline-rich protein 2G	5.61	0.00024	Y
	*Lce3c*	late cornified envelope 3C	5.03	1.21E-07	Y
	*Sprr2a3*	small proline-rich protein 2A3	4.65	0.00341	Y
	*Sprr2f*	small proline-rich protein 2F	4.31	2.76E-03	Y
	*Sprr2e*	small proline-rich protein 2E	3.69	0.14988	Y
	*Lce1e*	late cornified envelope 1E	3.3	4.99E-07	Y
	*Lce1k*	late cornified envelope 1K	3.22	6.24E-06	Y
	*Sprr1a*	small proline-rich protein 1A (Cornifin A)	3	6.86E-02	Y
	*Sprr4*	small proline-rich protein 4	2.92	0.35494	Y
	*Lce1f*	late cornified envelope 1F	2.85	2.76E-06	Y
	*Lce6a*	late cornified envelope 6A	2.64	2.36E-07	Y
	*Lce1j*	late cornified envelope 1J	2.18	2.34E-03	Y
	*Ivl*	involucrin	2	1.96E-11	Y
	*Tgm7*	transglutaminase 7	−7.40	5.37E-06	N
**Genes annotated to keratinization** **(GO:0031424) (*p*-value = 2.100e-5)** **and Keratin gene expression**	*Krt16*	keratin 16	7.53	1.74E-05	N
	*Krt42*	keratin 42	7.00	2.59E-05	N
	*Krt6b*	keratin 6B	6.56	0.042	N
	*Krt79*	keratin 79	6.51	0.00052	N
	*Krt6a*	keratin 6A	5.75	0.00054	N
	*Casp14*	caspase 14	3.51	4.26E-11	N
	*Krt17*	keratin 17	3.47	9.06E-09	N
	*Tgm3*	transglutaminase 3, E polypeptide	3.44	5.11E-10	N
	*Krt7*	keratin 7	3.19	2.10E-08	N
	*Hrnr*	hornerin	2.97	1.34E-05	Y
	*Krt4*	keratin 4	2.39	9.26E-03	N
	*Krt 14*	keratin 14	1.97	2.97E-05	N
	*Cdh3*	cadherin 3	1.61	0.0017	N
	*Krt 24*	keratin 24	-5.47	1.84E-07	N
	*Krt 15*	keratin 15	-3.33	4.13E-09	N

## Data Availability

The data discussed in this publication have been deposited in the NCBI’s Gene Expression Omnibus (GEO) [97] and are accessible through GEO Series accession number GSE164024 (https://www.ncbi.nlm.nih.gov/geo/query/acc.cgi?acc=GSE164024).

## Supplementary Materials

The following are available online at https://www.mdpi.com/article/10.3390/ijms22168634/s1.
