# Supplementary material for: Epidermal PPARγ Is a Key Homeostatic Regulator of Cutaneous Inflammation and Barrier Function in Mouse Skin
Source: Int J Mol Sci. 2021 Aug 11;22(16):8634. doi: 10.3390/ijms22168634 (PMC8395473; doi:10.3390/ijms22168634)
Supplement: Supplementary file 1 [file ijms-22-08634-s001.zip › Konger Supplemental 2nd revision.pdf]

Supplemental Information

Epidermal PPARγ is a key homeostatic regulator of cutaneous inflammation and barrier function in mouse skin.

Raymond L. Konger, Ethel Derr-Yellin, Teresa A Zimmers, Terrence Katona, Xiaoling Xuei, Yunlong Liu, Hong-Ming Zhou, Ed Ronald Simpson Jr, and Matthew J. Turner.

Table of Contents:

Fig S1

Fig S2

Supplemental Table 2

Supplemental Table 3

Fig S3

Supplemental Table 4

Supplemental Table 5

Supplemental Table 6

Fig S4

Fig S5

Fig S6

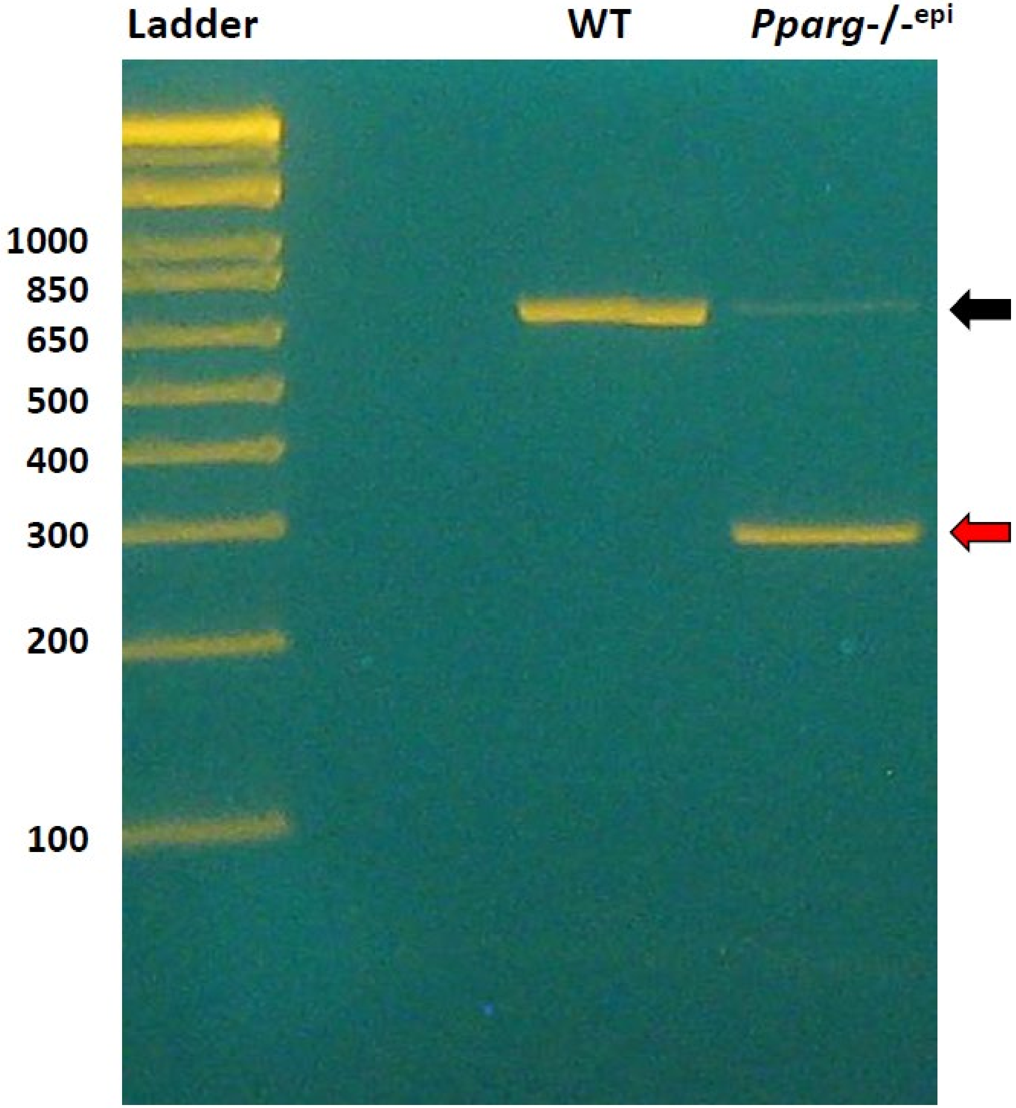

Fig S1: Detection of WT and mutant Pparg1 mRNA in RNA prepared from cultured keratinocytes. Wildtype (WT) and *Pparg-/-* ^{epi} mice were euthanized and the dorsal epidermis was removed for keratinocyte culture. Total RNA was extracted, and RT-PCR performed using primers that detect the WT transcript (700 bp, black arrow) or mutant transcript (300 bp, red arrow).

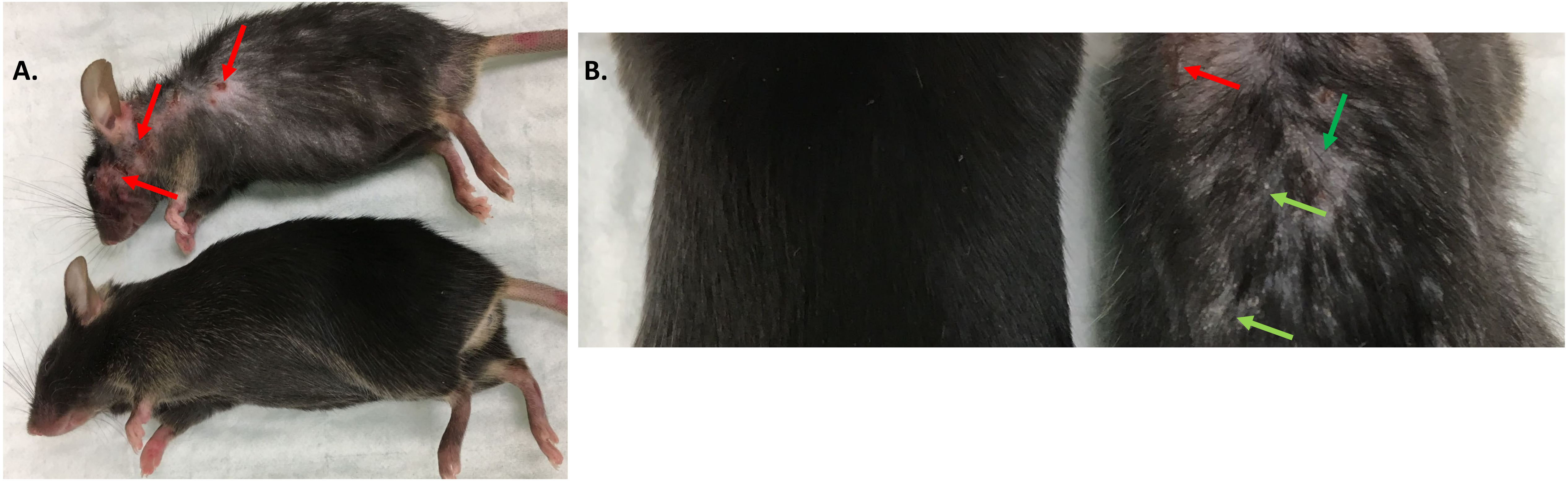

Fig S2.

A.) Compared to a wildtype control (bottom mouse), C57.*Pparg-/-*^{epi} mice (top mouse) have sparse hair (alopecia) and areas of inflammation and erosions (red arrows).

B.) Compared to a wildtype control (left mouse), C57.*Pparg-/-*^{epi} mice (right mouse) have an area of excoriation (red arrow), with alopecia and flaky hyperkeratotic skin with dandruff (green arrows).

Supplemental Table 2: Phenotype of wildtype (WT) sibling controls & C57.*Pparg-/-*^{epi} mice

Phenotype		Hair loss			Dandruff			Blepharitis			Erythematous lesions			Skin wounds***		
Age (Weeks)	Genotype	Yes	No	P-value*	Yes	No	P-value*	Yes	No	P-value*	Yes	No	P-value*	Yes	No	P-value*
18-23	WT	0	6	p=0.0003	0	6	p=0.0003	0	6	p=0.0003	0	6	p=0.0003	0	6	p=0.0097
	Pparg-/-^{epi}	8	0		8	0		8	0		8	0		6	2	
8-10	WT	0	8	p<0.0001	0	8	p<0.0001	0	8	p<0.0001	0	8	p<0.0001	0	8	p=1.0000
	Pparg-/-^{epi}	12	1		12	1		12	1		12	1		1	12	
3-4	WT	0	14	p<0.0001	0	14	p<0.0001	0	14	p<0.0001	0	14	p<0.0001	0	14	NA**
	Pparg-/-^{epi}	14	1		12	3		13	2		12	3		0	15	

* Fisher's exact test (two-sided)
** Unable to calculate with one row filled with zeros
*** Erosions and excoriations

Supplemental Table 3: Cytokine-cytokine receptor interaction - Mus musculus KEGG: 04060

|Log2fc| > 0.6 AND FDR < 0.05

#	symbol	entrez	log2fc	fold change	FDR
1	*Il1f6*	54448	3.90	14.88	0.000001
2	*Cxcr2*	12765	3.53	11.58	0.000099
3	*Ccl1*	20290	3.37	10.31	0.000001
4	*Ccr1*	12768	3.27	9.62	0.001382
5	*Il23a*	83430	3.05	8.29	1.55E-05
6	*Inhba*	16323	2.70	6.49	0.000001
7	*Ccl2*	20296	2.49	5.62	8.37E-05
8	*Il21r*	60504	2.35	5.09	0.000001
9	*Cxcl9*	17329	2.17	4.49	0.0009
10	*Ccr6*	12458	2.15	4.44	0.01565
11	*Il1f8*	69677	2.11	4.30	0.000001
12	*Il12b*	16160	2.09	4.27	0.00026
13	*Cxcl10*	15945	2.07	4.19	0.00157
14	*Ccr7*	12775	2.07	4.19	0.000157
15	*Inhbb*	16324	1.90	3.73	0.0399
16	*Tnfsf13*	69583	1.87	3.65	1.34E-05
17	*Ccl20*	20297	1.80	3.49	0.00937
18	*Ccr5*	12774	1.79	3.45	0.025535
19	*Tnfsf9*	21950	1.74	3.34	0.000104
20	*Ccl22*	20299	1.65	3.13	4.68E-06
21	*Il9r*	16199	1.56	2.95	0.025078
22	*Il1b*	16176	1.56	2.94	0.0271
23	*Tnfrsf9*	21942	1.53	2.89	5.09E-05
24	*Ccl7*	20306	1.50	2.83	0.0148
25	*Cxcr4*	12767	1.45	2.73	0.017978
26	*Ccl19*	24047	1.41	2.66	0.013322
27	*Il18rap*	16174	1.32	2.49	0.000143
28	*Csf2rb2*	12984	1.30	2.47	2.31E-05
29	*Ccl17*	20295	1.27	2.41	0.016702
30	*Cxcl16*	66102	1.18	2.27	0.000001
31	*Bmp7*	12162	1.18	2.26	8.93E-05
32	*Il27ra*	50931	1.15	2.21	0.025203
33	*Csf3r*	12986	1.13	2.19	0.0407
34	*Tnfsf15*	326623	1.13	2.19	0.000455
35	*Csf2rb*	12983	1.04	2.05	0.00841
36	*Tnfrsf12a*	27279	1.03	2.04	0.00258
37	*Il18*	16173	1.01	2.01	8.71E-05
38	*Il2ra*	16184	1.00	2.00	0.0204
39	*Tnf*	21926	0.92	1.89	0.0251
40	*Il1f9*	215257	0.88	1.85	0.000601
41	*Tnfrsf1b*	21938	0.88	1.84	0.0154
42	*Cxcr6*	80901	0.75	1.68	0.015752
43	*Il1f5*	54450	0.70	1.63	0.000333
44	*Il4ra*	16190	0.67	1.59	0.000734
45	*Bmp2*	12156	-0.68	-1.61	0.030541
46	*Ackr4*	252837	-0.76	-1.69	0.000157
47	*Il18r1*	16182	-0.78	-1.72	0.001735
48	*Il33*	77125	-0.86	-1.81	0.009105
49	*Il1r2*	16178	-0.94	-1.92	0.00209
50	*Tnfrsf21*	94185	-0.96	-1.95	3.72E-06
51	*Il15*	16168	-1.02	-2.03	0.009471
52	*Ccr4*	12773	-1.03	-2.04	0.007785
53	*Il34*	76527	-1.03	-2.05	2.48E-05
54	*Bmp4*	12159	-1.12	-2.17	7.04E-05
55	*Fas*	14102	-1.13	-2.18	1.64E-05
56	*Tnfrsf13c*	72049	-1.15	-2.21	0.0114
57	*Ngfr*	18053	-1.21	-2.31	0.000157
58	*Il12rb2*	16162	-1.29	-2.45	0.000001
59	*Cxcl14*	57266	-1.38	-2.61	0.00334
60	*Tnfrsf11b*	18383	-1.46	-2.75	0.000001
61	*Tnfsf18*	240873	-1.53	-2.89	0.00435
62	*Il20ra*	237313	-1.54	-2.91	7.22E-05
63	*Il31ra*	218624	-2.99	-7.96	0.000589

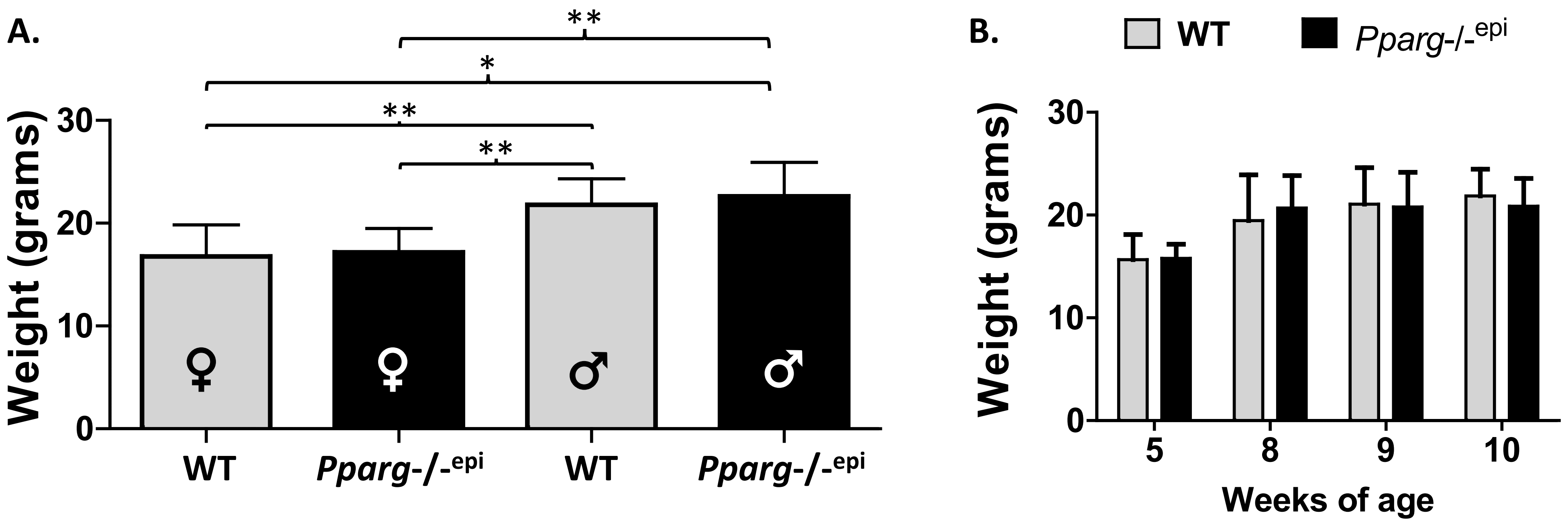

Fig S3. Mouse weights as a function of gender, age and knockout status. A.) Male (♂) mice are larger than female (♀) mice, but the absence of epidermal *Pparg* has no effect on mouse weight for either gender. C57.*Pparg*-/-^{epi} (*Pparg*-/-^{epi}) and their wildtype (WT) sibling controls were age-matched from from the range of 5-10 weeks of age to determine whether loss of epidermal Pparg or gender were associated with a change in weight (n=5 mice per group, age-matched at a mean of 7.4 weeks of age per group). Male mice of both genotypes were significantly larger than the female mice of either genotype. There was no significant difference in mouse weights between WT and *Pparg*-/-^{epi} mice regardless of gender. *, $p<0.05$; **, $p<0.01$; One-way ANOVA with Tukey's multiple comparisons test. **B.) There is no significant difference in WT and *Pparg*-/-^{epi} mouse weight at any week in the period of 5 to 10 weeks of age.** Male and female mice for each genotype were weighed. A total of 13 (week 5), 17 (week 8), 16 (week 9) and 9 (week 10) male and female mice of both genotypes were weighed. The mean weight per gender and genotype was then determined. The results represent the average of the male and female mean weights for each age group.

A.

Pruning Type: None				Pruning Type: Elim		Pruning Type: Weight	
GO Term	p-value	p-value (FDR)	p-value (Bonferroni)	GO Term	p-value	GO Term	p-value
cytokine activity	5.100e-14	9.320e-11	9.506e-11	cytokine activity	1.300e-9	cytokine activity	5.100e-14
transmembrane signaling receptor activity	1.000e-13	9.320e-11	1.863e-10	serine-type endopeptidase activity	5.800e-8	serine-type endopeptidase activity	5.800e-8
receptor ligand activity	4.700e-13	2.920e-10	8.761e-10	calcium ion binding	1.000e-6	cytokine receptor activity	3.100e-7
signaling receptor activity	1.200e-12	5.591e-10	2.237e-9	G protein-coupled receptor activity	4.500e-6	calcium ion binding	1.000e-6
receptor regulator activity	6.300e-12	2.349e-9	1.174e-8	chemokine activity	5.200e-6	G protein-coupled receptor activity	1.200e-5

B.

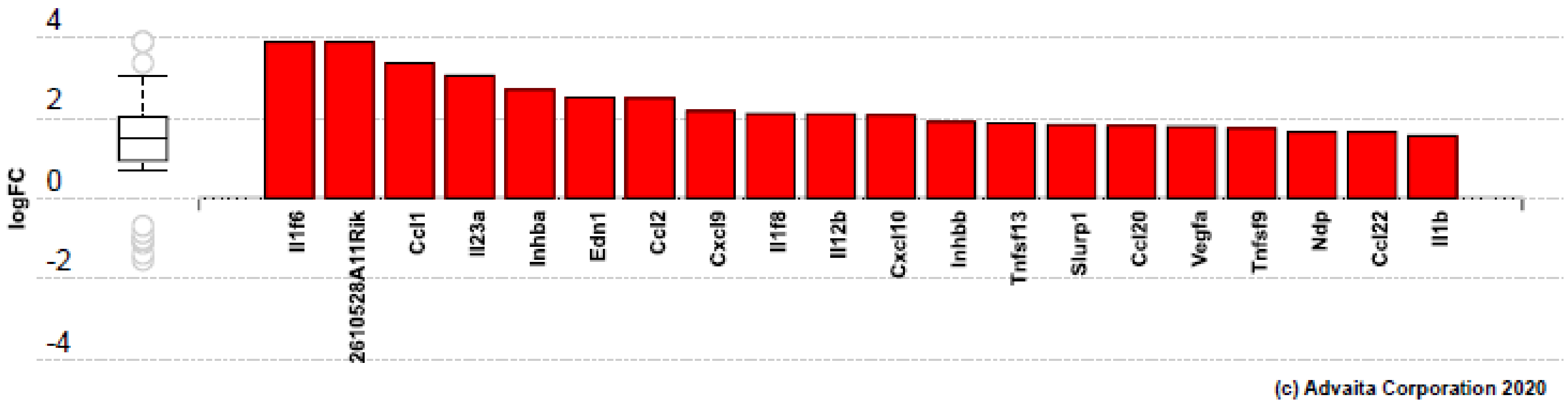

Fig S4.

A.) After mRNA sequencing of wildtype and C57.*Pparg-/-*^{epi} mouse epidermal scrapings, differential expression (DE) analysis was performed and Gene Ontology (GO) analysis was performed for Molecular Function GO terms (Advaita Corp). The top 5 GO groups that annotate to the DE data are shown after no pruning, Elim or Weight pruning.

B.) All the differentially expressed genes that are annotated to cytokine activity (GO:0005125) are ranked based on their value of log2 fold change. The plot shows the top 20 of 42 differentially expressed genes. The box and whisker plot on the left summarizes the distribution of all the differentially expressed genes that are annotated to this GO term. The box represents the 1st quartile, the median and the 3rd quartile, while the outliers are represented by circles.

Supplemental Table 4: Cytokine activity GO:0005125

			|Log2fc| > 0.6 AND FDR < 0.05							|Log2fc| > 0.6 AND FDR < 0.05		
#	symbol	entrez	log2fc	fold change	FDR		#	symbol	entrez	log2fc	fold change	FDR
1	*Il1f6*	54448	3.90	14.88	0.000001		22	*Ccl7*	20306	1.50	2.83	0.0148
2	*2610528A11Rik*	70045	3.89	14.84	0.000001		23	*Ccl19*	24047	1.41	2.66	0.013322
3	*Ccl1*	20290	3.37	10.31	0.000001		24	*Ebi3*	50498	1.31	2.48	0.007295
4	*Il23a*	83430	3.05	8.29	1.55E-05		25	*Ccl17*	20295	1.27	2.41	0.016702
5	*Inhba*	16323	2.70	6.49	0.000001		26	*Pglyrp1*	21946	1.27	2.41	0.00434
6	*Edn1*	13614	2.51	5.70	0.000001		27	*Cxcl16*	66102	1.18	2.27	0.000001
7	*Ccl2*	20296	2.49	5.62	8.37E-05		28	*Bmp7*	12162	1.18	2.26	8.93E-05
8	*Cxcl9*	17329	2.17	4.49	0.0009		29	*Tnfsf15*	326623	1.13	2.19	0.000455
9	*Il1f8*	69677	2.11	4.30	0.000001		30	*Kitl*	17311	1.12	2.18	0.0112
10	*Il12b*	16160	2.09	4.27	0.00026		31	*Il18*	16173	1.01	2.01	8.71E-05
11	*Cxcl10*	15945	2.07	4.19	0.00157		32	*Tnf*	21926	0.92	1.89	0.0251
12	*Inhbb*	16324	1.90	3.73	0.0399		33	*Il1f9*	215257	0.88	1.85	0.000601
13	*Tnfsf13*	69583	1.87	3.65	1.34E-05		34	*Il1f5*	54450	0.70	-1.63	0.000333
14	*Slurp1*	57277	1.83	3.55	1.16E-05		35	*Bmp2*	12156	-0.68	-1.61	0.030541
15	*Ccl20*	20297	1.80	3.49	0.00937		36	*Il33*	77125	-0.86	-1.81	0.009105
16	*Vegfa*	22339	1.78	3.43	0.000001		37	*Il15*	16168	-1.02	-2.03	0.009471
17	*Tnfsf9*	21950	1.74	3.34	0.000104		38	*Crlf1*	12931	-1.02	-2.03	0.022838
18	*Ndp*	17986	1.65	3.14	0.048804		39	*Il34*	76527	-1.03	-2.05	2.48E-05
19	*Ccl22*	20299	1.65	3.13	4.68E-06		40	*Bmp4*	12159	-1.12	-2.17	7.04E-05
20	*Il1b*	16176	1.56	2.94	0.0271		41	*Cxcl14*	57266	-1.38	-2.61	0.00334
21	*Timp1*	21857	1.51	2.86	0.008624		42	*Tnfsf18*	240873	-1.53	-2.89	0.00435

Supplemental Table 5: Antimicrobial humoral response - Mus musculus GO: 0019730					
			│Log2fc│ > 0.6 AND FDR < 0.05		
#	symbol	Gene name	log2fc	fold change	FDR
1	*Npy*	neuropeptide Y	3.47091	11.08785	0.000606876
2	*Ccl1*	chemokine (C-C motif) ligand 1	3.36665	10.31486	7.67E-08
3	*S100a9*	S100 calcium binding protein A9 (calgranulin B)	2.72042	6.59067	8.00E-06
4	*Krt6a*	keratin 6A	2.52339	5.74933	0.000535161
5	*Slpi*	secretory leukocyte peptidase inhibitor	2.50067	5.65947	7.98E-05
6	*Ccl2*	chemokine (C-C motif) ligand 2	2.48946	5.61568	8.37E-05
7	*Cxcl9*	chemokine (C-X-C motif) ligand 9	2.16677	4.49016	0.000899589
8	*Cxcl10*	chemokine (C-X-C motif) ligand 10	2.06832	4.19397	1.57E-03
9	*Bcl3*	B cell leukemia/lymphoma 3	1.90686	3.74992	9.14E-08
10	*Ccl20*	chemokine (C-C motif) ligand 20	1.80131	3.48536	9.37E-03
11	*Ang2*	angiogenin, ribonuclease A family, member 2	1.75091	3.36571	4.22E-02
12	*Ccl22*	chemokine (C-C motif) ligand 22	1.64502	3.12753	4.68E-06
13	*Nod2*	nucleotide-binding oligomerization domain containing 2	1.48099	2.79140	8.76E-07
14	*Colec11*	collectin sub-family member 11	1.45847	2.74816	2.90E-02
15	*Ccl19*	chemokine (C-C motif) ligand 19	1.41034	2.65800	0.013322181
16	*Ccl17*	chemokine (C-C motif) ligand 17	1.27182	2.41465	0.016702333
17	*Pglyrp1*	peptidoglycan recognition protein 1	1.26689	2.40643	4.34E-03
18	*Pglyrp3*	peptidoglycan recognition protein 3	1.00304	2.00422	1.51E-03
19	*Gapdh*	glyceraldehyde-3-phosphate dehydrogenase	0.83684	1.78613	1.36E-02
20	*Il1f5*	interleukin 1 family, member 5 (delta)	0.70193	1.62668	3.33E-04
21	*Jchain*	immunoglobulin joining chain	-0.665	-1.58557	0.657815763
22	*Cxcl14*	chemokine (C-X-C motif) ligand 14	-1.3842	-2.61024	3.34E-03
23	*Wfdc3*	WAP four-disulfide core domain 3	-3.4483	-10.91538	1.86E-03

Supplemental Table 6: Pyroptosis - Mus musculus GO: 0070269					
			│Log2fc│ > 0.6 AND FDR < 0.05		
#	symbol	Gene name	log2fc	fold change	FDR
1	*Gsdma2*	gasdermin A2	4.098255464	17.12765	1.16E-05
2	*Gsdmc*	gasdermin C	2.830369448	7.11256	1.95E-07
3	*Gsdma3*	gasdermin A3	1.910314644	3.75891	1.24E-01
4	*Aim2*	absent in melanoma 2	1.876486072	3.67180	1.11E-18
5	*Gsdmc2*	gasdermin C2	1.269615027	2.41097	1.46E-02
6	*Casp4*	caspase 4, apoptosis-related cysteine peptidase	1.121450935	2.17566	1.57E-07
7	*Casp1*	caspase 1	1.104419053	2.15012	1.09E-06
8	*Gsdma*	gasdermin A	1.065759185	2.09327	2.24E-09
9	*Nlrp1b*	NLR family, pyrin domain containing 1B	-1.410396194	-2.65810	1.02E-06

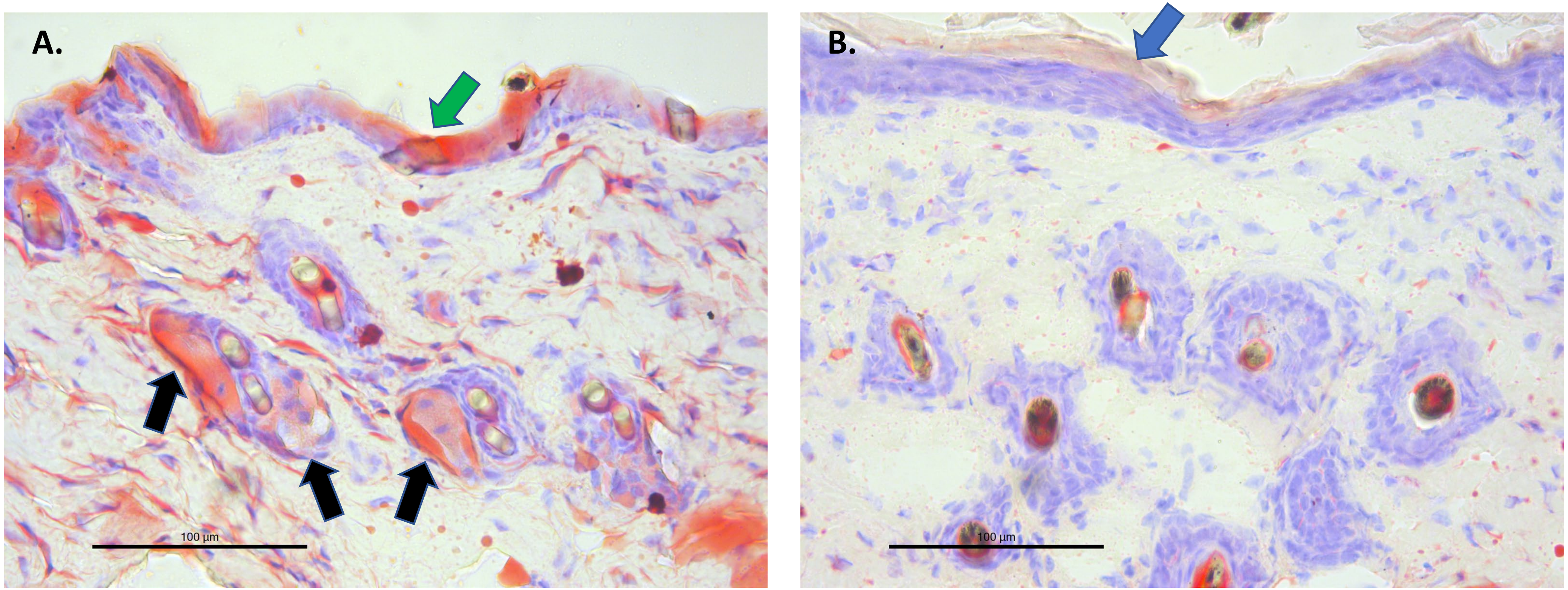

Fig S5: C57.Pparg-/-^{epi} mouse skin lacks stratum corneum lipid and evidence of lipid-laden sebocytes. Wildtype and C57.*Pparg*-/-^{epi} mouse skin was stained with Oil Red O to assess the lipid content in sebaceous glands and the stratum corneum. **A.)** Wildtype skin showed staining in the stratum corneum (green arrow) that is consistent with an intact lipid barrier. Staining of sebaceous glands is also observed surrounding hair follicles (black arrows). **B.)** C57.*Pparg*-/-^{epi} skin shows hyperkeratosis with a relative paucity of lipid staining in the stratum corneum (blue arrow). Some lipid staining is observed in the hair shaft of hair follicles, but the absence of holocrine glands containing sebaceous cells loaded with sebaceous lipids. Bar represent 100 µm.

Fig S6: PPARγ immuno-fluorescent staining (IF) shows prominent sebaceous gland immuno-labeling. IF labeling of PPARγ (Red), pan-cytokeratin (Green) and nuclear labeling with DAPI (Blue) was performed. A-C). Representative 400x photo-micrographs of *Pparg-/-*[epi] skin. (A) Grescale image of PPARγ staining. (B) Greyscale image of DAPI staining. (C). Merged image with DAPI (blue), PPARγ (red) and pan-cytokeratin (green). D-F)

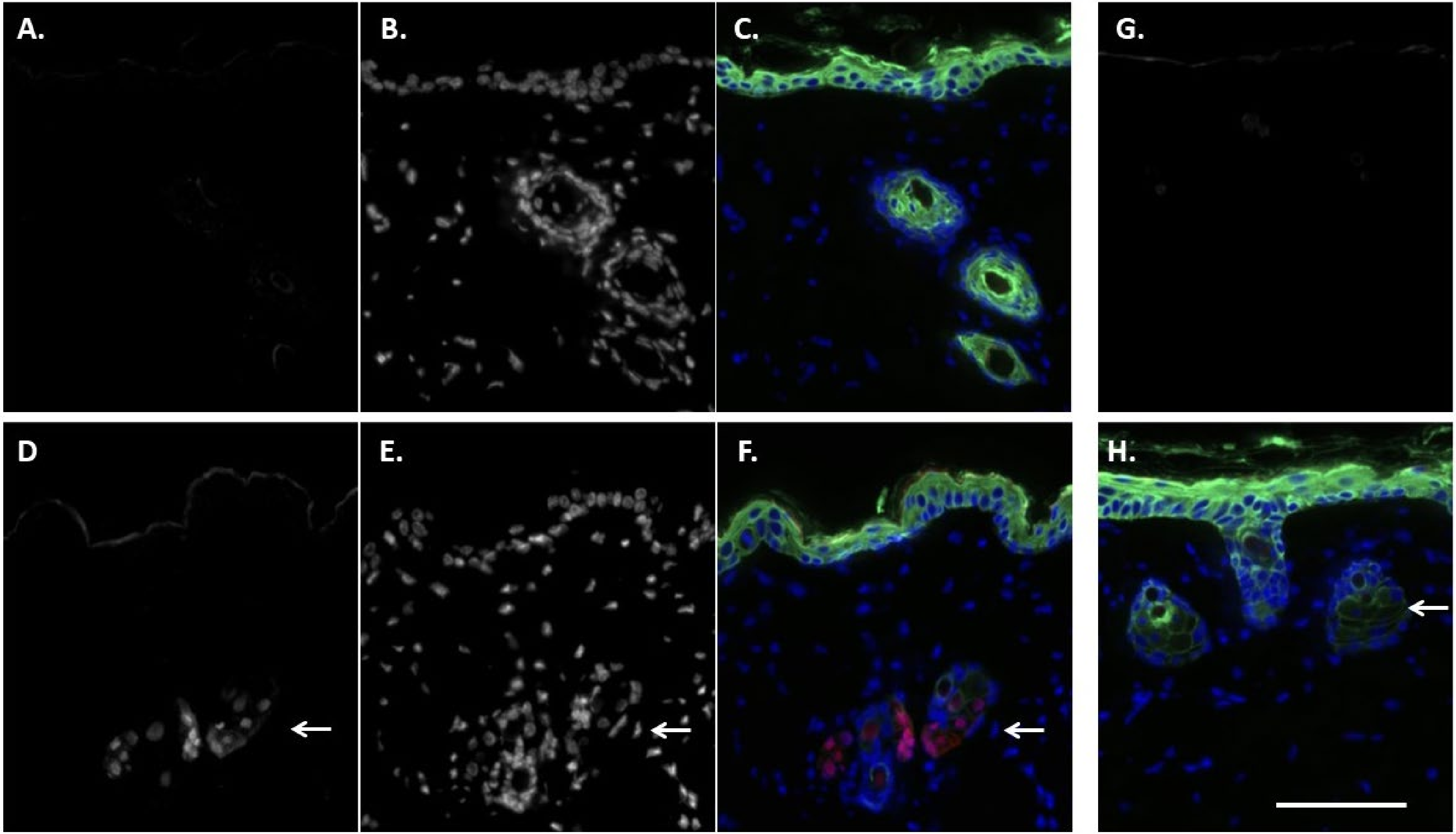

Representative 400x images of wildtype (WT) mouse skin. (A) Grescale image of PPARγ staining. (B) Greyscale image of DAPI staining. (C). Merged image with DAPI (blue), PPARγ (red) and pan-cytokeratin (green). G.) Greyscale image of WT skin negative control (no PPARγ primary). H.) Merged image of WT skin negative control. Arrows point to areas with sebaceous glands. Bar in lower right of panel H = 100 μm.

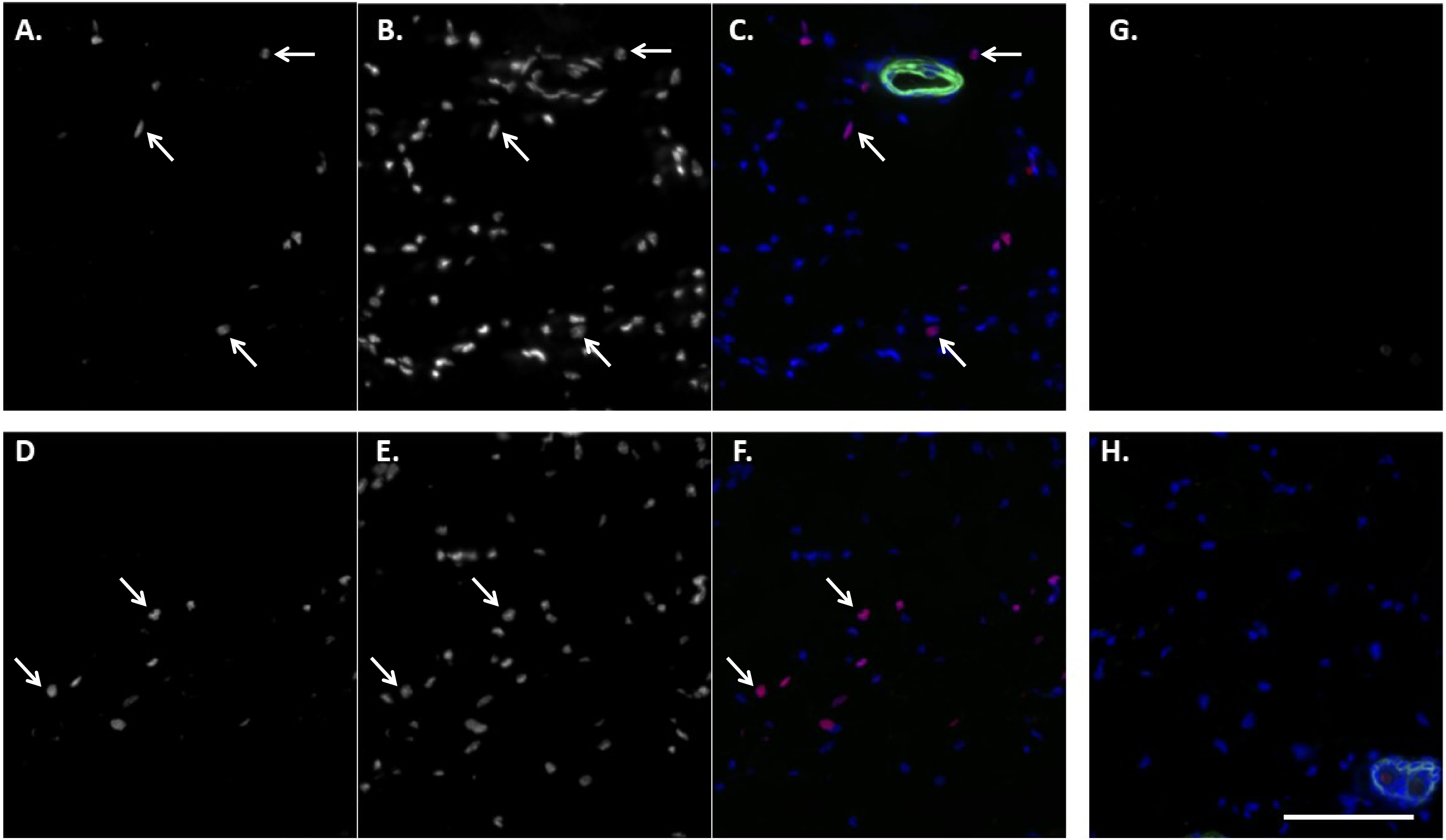

Fig S7: PPARγ immuno-fluorescent staining (IF) of hypodermal fat shows prominent nuclear immuno-labeling. IF labeling of PPARγ (Red), pan-cytokeratin (Green) and nuclear labeling with DAPI (Blue) was performed. A-C). Representative 400x photo-micrographs of *Pparg-/-*[epi] hypodermal fat. (A) Grescale image of PPARγ staining. (B) Greyscale image of DAPI staining. (C). Merged image with DAPI (blue), PPARγ (red) and pan-cytokeratin (green). D-F) Representative 400x images of hypodermal adipose tissue in wildtype (WT) mouse skin. (A) Grescale image of PPARγ staining. (B) Greyscale image of DAPI staining. (C). Merged image with DAPI (blue), PPARγ (red) and pan-cytokeratin (green). G.) Greyscale image of WT skin negative control (no PPARγ primary). H.) Merged image of WT skin negative control. Arrows point to several PPARγ+ nuclei within the hypodermal fat. Bar in lower right of panel H = 100 µm.
